# Supplementary material for: Investigating the feasibility and acceptability of the HOLOBalance system compared with standard care in older adults at risk for falls: study protocol for an assessor blinded pilot randomised controlled study
Source: BMJ Open. 2021 Feb 12;11(2):e039254. doi: 10.1136/bmjopen-2020-039254 (PMC7883859; doi:10.1136/bmjopen-2020-039254)
Supplement: Author's manuscript [file bmjopen-2020-039254.draft_revisions.pdf]

BMJ Open is committed to open peer review. As part of this commitment we make the peer review history of every article we publish publicly available.

When an article is published we post the peer reviewers' comments and the authors' responses online. We also post the versions of the paper that were used during peer review. These are the versions that the peer review comments apply to.

The versions of the paper that follow are the versions that were submitted during the peer review process. They are not the versions of record or the final published versions. They should not be cited or distributed as the published version of this manuscript.

BMJ Open is an open access journal and the full, final, typeset and author-corrected version of record of the manuscript is available on our site with no access controls, subscription charges or pay-per-view fees (http://bmjopen.bmj.com).

If you have any questions on BMJ Open's open peer review process please email info.bmjopen@bmj.com

BMJ Open

Investigating the Feasibility and Acceptability of the HOLOBalance System Compared to Standard Care in Older Adults at Risk for Falls: Study protocol for an assessor blinded pilot randomised-controlled study.

Journal:	*BMJ Open*
Manuscript ID	bmjopen-2020-039254
Article Type:	Protocol
Date Submitted by the Author:	08-Apr-2020
Complete List of Authors:	Liston, Matthew; King's College London, Centre for Human and Applied Physiological Sciences<br>Genna, Gregory; University College London,<br>Maurer, Christoph; University of Freiburg, Department of Neurology and Neuroscience, Medical Center,<br>Kikidis, Dimitris; National and Kapodistrian University of Athens<br>Gatsios, Dimitris; University of Ioannina, Unit of Medical Technology and Intelligent Information Systems, Department of Material Science and Engineering; University of Ioannina, Department of Neurology, Medical School,<br>Fotiadis, Dimitris; University of Ioannina, Unit of Medical Technology and Intelligent Information Systems, Department of Material Science and Engineering<br>Bamiou, Doris-Eva; University College London, The Ear Institute;<br>Pavlou, Marousa; Kings Coll London, Centre for Human and Applied Physiological Sciences
Keywords:	GERIATRIC MEDICINE, REHABILITATION MEDICINE, Telemedicine < BIOTECHNOLOGY & BIOINFORMATICS

SCHOLARONE™
Manuscripts

I, the Submitting Author has the right to grant and does grant on behalf of all authors of the Work (as defined in the below author licence), an exclusive licence and/or a non-exclusive licence for contributions from authors who are: i) UK Crown employees; ii) where BMJ has agreed a CC-BY licence shall apply, and/or iii) in accordance with the terms applicable for US Federal Government officers or employees acting as part of their official duties; on a worldwide, perpetual, irrevocable, royalty-free basis to BMJ Publishing Group Ltd ("BMJ") its licensees and where the relevant Journal is co-owned by BMJ to the co-owners of the Journal, to publish the Work in this journal and any other BMJ products and to exploit all rights, as set out in our licence.

The Submitting Author accepts and understands that any supply made under these terms is made by BMJ to the Submitting Author unless you are acting as an employee on behalf of your employer or a postgraduate student of an affiliated institution which is paying any applicable article publishing charge ("APC") for Open Access articles. Where the Submitting Author wishes to make the Work available on an Open Access basis (and intends to pay the relevant APC), the terms of reuse of such Open Access shall be governed by a Creative Commons licence – details of these licences and which Creative Commons licence will apply to this Work are set out in our licence referred to above.

Other than as permitted in any relevant BMJ Author's Self Archiving Policies, I confirm this Work has not been accepted for publication elsewhere, is not being considered for publication elsewhere and does not duplicate material already published. I confirm all authors consent to publication of this Work and authorise the granting of this licence.

For peer review only

Investigating the Feasibility and Acceptability of the HOLOBalance System Compared to Standard Care in Older Adults at Risk for Falls: Study protocol for an assessor blinded pilot randomised-controlled study.

Matthew B Liston[1], Gregory J Genna[2], Christoph Maurer[3], Dimitris Kikidis[4], Dimitris Gatsios[5,6,] Dimitris Fotiadis, Doris Eva-Bamiou[2,7,8], Marousa Pavlou[1].

[1] Centre for Human and Applied Physiological Sciences, King's College London. UK
[2]The Ear Institute, University College London, London, UK
[3] Department of Neurology and Neuroscience, Medical Center, University of Freiburg, Freiburg, Germany
[4] Department of Otolaryngology, National and Kapodistrian University of Athens, Athens, Greece
[5]Department of Neurology, Medical School, University of Ioannina, Ioannina, Greece
[6]Unit of Medical Technology and Intelligent Information Systems, Department of Material Science and Engineering, University of Ioannina, Ioannina, Greece
[7] Neuro-Otology Department, University College London Hospitals, London, UK
[8]Biomedical Research Centre, National Institute for Health Research, London, UK

CORRESPONDING AUTHOR:

Liston, Matthew B. Centre for Human and Applied Physiological Sciences, King's College London. London, United Kingdom. SE1 1UL. Matthew.Liston@kcl.ac.uk

WORD COUNT

3979 words

PROTOCOL VERSION: Version 2, 20th January 2020

For peer review only - http://bmjopen.bmj.com/site/about/guidelines.xhtml

ABSTRACT

Introduction: Approximately 1/3 of all older adults fall each year, with wide ranging physical, psychosocial and healthcare related consequences. Strength and balance training is the cornerstone for falls prevention programmes, yet these are not consistently provided, do not routinely address all components of the balance system, and are often not well attended. The HOLOBalance system provides an evidence-based balance training programme delivered to patients in their home environment using a novel technological approach including an augmented reality virtual physiotherapist, exergames and a remote monitoring system. The aims of this proof of concept study are to 1) determine the safety, acceptability and feasibility of providing HOLOBalance to community dwelling older adults at risk for falls and 2) provide data to support sample size estimates for a future trial should trends for effectiveness be identified.

Methods: A single (assessor) blinded pilot randomised controlled proof of concept study. 120 participants will be randomised to receive an 8-week home exercise programme consisting of either: 1) HOLOBalance, or 2) The OTAGO Home Exercise Programme. Participants will be required to complete their exercise programme independently under the supervision of a physiotherapist. Participants will have weekly telephone contact with their physiotherapist, and will receive home visits at weeks 0, 3 and 6. Outcome measures of safety, acceptability and feasibility, clinical measures of balance function, disability, balance confidence and cognitive function will be assessed before and immediately after the 8 week intervention. Acceptability and feasibility will be explored using descriptive statistics, and trends for effectiveness will be explored using GLM ANOVA.

Ethics and Dissemination: This study has received institutional ethical approvals in Germany (reference: 265/19), Greece (reference: 9769/24-6-2019) and the UK (reference: 19/LO/1908). Findings from this study will be submitted for peer reviewed publications.

Trial Registration Number: NCT04053829.

Keywords: Older Adults, Falls Prevention, Falls Rehabilitation, Tele-rehabilitation, Protocol.

STRENGTHS AND LIMITATIONS OF THIS STUDY

The HOLOBalance telerehabilitation programme addresses all facets of the postural control system and provides an individualised training programme prescribed by a treating clinician. The addition of exergames will improve user engagement and remote monitoring will allow for clinicians to track participant progress.

For peer review only - http://bmjopen.bmj.com/site/about/guidelines.xhtml

This is the first study to explore the acceptability and feasibility of providing holographic balance training programmes to older adults at risk for falls in their home environment. This will provide crucial information for the future implementation of these novel technologies.

The HOLOBalance study is not powered to determine the effect of the intervention on falls risk or falls rate. A future definitive trial will be required to determine efficacy of the intervention.

For peer review only

For peer review only - http://bmjopen.bmj.com/site/about/guidelines.xhtml

INTRODUCTION

One in three people over the age of 65 fall annually, with the majority of these being caused by balance dysfunction [1, 2]. Maintaining balance is complex and depends upon sensory inputs from the visual, proprioceptive and vestibular systems to provide information on body position and motion. Alongside this, central processes monitor and control the interaction between musculoskeletal and neural systems to generate anticipatory postural adjustments and adapt posture to changing environmental and balance task demands [3]. Age-related decline in these systems, are well documented and lead to impaired postural and ambulatory control thus increasing risk for falls [4, 5]. Recent studies have identified that inner ear balance dysfunction is common in older adults who fall [4, 6-11], with up to 80% of fallers experiencing vestibular dysfunction [7]. Although sensory inputs are crucial for postural control, cognitive functions are equally as important for maintaining safe community ambulation. Deficits in executive function and attention are common in older age and are associated with impairments in postural control, dual-tasking balance ability (i.e. walk and talk at the same time), reduced gait speed and increased falls risk [12-18]. Additionally, changes in cognitive function strongly predict impairment in activities of daily living and functional independence [17, 19-21]. Thus, falls must be considered as multi-factorial in nature and should be targeted in interventions that address domains that impact upon balance and quality of life.

As falls have wide-ranging physical and psychological consequences and increase the likelihood of frailty, cognitive decline, sedentary behaviour, social exclusion, injury and death [1-5, 19, 22], their prevention is of primary concern to many public health bodies [23-25]. The National Institute of Clinical Excellence (NICE) UK Guidelines [26] recommend that older adults at risk for falls should undergo a balance assessment and receive targeted interventions to improve balance and reduce risk for falls. Despite the strength of available evidence, compliance and implementation for providing balance physiotherapy have been poor in UK healthcare settings [27].

Balance Physiotherapy typically consists of personalized sets of exercises, defined by a healthcare professional and is the only effective treatment for balance disorders, irrespective of a person's age [28-30]. Although exercises are brief and easy to perform, there is up to 50% loss in follow up rate in older adults in these programmes, and while supervision significantly increases compliance and effectiveness [31], it is costly to provide. To this end, many falls prevention programmes provide exercise programmes completed either in group settings or independently at home [32-37]. These programmes have been shown to be effective in reducing falls rates in older adults, reducing falls by 30-40% (dependant on target

For peer review only - http://bmjopen.bmj.com/site/about/guidelines.xhtml

population), however they are limited in that they either do not address inner ear related balance function or dual task training [33-35], or they do not address such aspects until very late in the programme [36, 37]. This is despite promising evidence suggesting that combined cognitive and functional training may provide improvements beyond single task training [38-40], since there has been limited carryover into clinical practice and falls prevention programmes. Similarly, rehabilitation programmes which address inner ear balance dysfunction have been developed for older adults at risk for falls and have shown substantial additional reduction of falls risk to standard programmes [41-43]. These programmes require customised, expert led interventions to optimise recovery, however there is limited availability of experts to provide these individualised interventions.

The HOLOBalance tele-rehabilitation platform has been developed in order to address these healthcare needs and to provide an interactive falls rehabilitation programme that incorporates functional balance training, exercises to improve inner ear balance function and cognitive-motor training within a comprehensive intervention that aims to improve balance function for older adults at risk for falls. HOLOBalance will use off-the-shelf technologies to allow clinicians to provide an individualised balance physiotherapy programme for older adults who will be able to participate in at a time of their choice and in their home environment (for system description please visit www.holobalance.eu). This study protocol outlines the multi-site randomised controlled proof of concept study to explore safety, feasibility and acceptability of the HOLOBalance tele-rehabilitation system.

For peer review only

METHODS AND ANALYSIS

Trial Design

This study is an assessor-blinded, randomised proof of concept study to explore the acceptability and feasibility of providing a home-based balance tele-rehabilitation programme (HOLOBalance) to community dwelling older adults at risk for falls. All participants will be enrolled in a 8-week home exercise programme designed to improve balance function, and which adheres to the current guidance for strength and balance training [26]. All participants will receive an exercise programme provided under the supervision of a physiotherapist with regular reviews by telephone (weekly) and home visits (Week 0,3 and 6). Exercises will be

For peer review only - http://bmjopen.bmj.com/site/about/guidelines.xhtml

prescribed according to participant feedback and task performance as assessed by the treating physiotherapist.

This study will compare acceptability of the HOLOBalance tele-health programme (e.g. compliance, drop-out rate) to an established home exercise programme that is routinely used in community rehabilitation (the OTAGO HEP [23, 79]) and will explore trends for effectiveness across a number of validated outcome measures to explore whether a future trial is warranted, and if so to provide data for a sample size estimate. For this proof of concept study, data will be collected at baseline (week 0) and at completion of the intervention (week 9). The flow of participants through the trial will be recorded in compliance with the CONSORT statement (figure 1), and this protocol has been developed using the SPIRIT reporting guidelines for clinical trials [44].

Participants

One hundred and twenty independently living, community dwelling older adults aged 65-80 who are at risk of falls will be recruited across three sites (London (UK), Freiberg (Germany), Athens (Greece)). Older adults who meet the following inclusion criteria for entry will be eligible to be enrolled.

Inclusion criteria

- Living independently in the community

- Able to walk 500 meters independently or with a stick

- Age 65 to 80 years

- At risk for falls (i.e. FGA less than 22/30), have significant fear of falling (FESI short form >10) or having experienced a fall/s in the last 12 months

- Not currently enrolled in a falls and/or cognitive rehabilitation programme

- No significant visual, musculoskeletal or neurological impairment

- No acute musculoskeletal injury that prevents participation in a structured exercise programme

- No orthostatic hypotension or uncontrolled hypertension

- No implanted medical devices e.g. cardiac pacemakers.

For peer review only - http://bmjopen.bmj.com/site/about/guidelines.xhtml

- No evidence of low mood (i.e. must score ≤10 on the Geriatric Depression Scale)

- A score of >22 on the MoCA, i.e. adults with no or mild cognitive impairment

- Willing to participate and to comply with the proposed training and testing regime.

- Available space of 1x2 metres at home and sufficient home broadband to allow the system to operate as designed

- Has not participated in a drug trial in the past 6 months

Recruitment

Community dwelling older adults will be recruited via adverts placed in the community, on social media, and by referrals made by recruiting clinics. Potential participants will undergo a telephone screening session with a researcher to determine their eligibility, with eligible participants booked in for baseline testing with the blinded outcome assessor. Participants will provide written informed consent on arrival at their baseline outcome assessment session.

Randomisation

Patients will be randomized using an online platform (www.sealedenvelope.com) into either the intervention or control groups. Participants will be allocated in blocks of 10 with an allocation ratio of 1:1 to control (OTAGO HEP) and intervention (HOLOBalance) groups. Randomisation procedures will be run independently for each site and will be completed by a researcher external to the research study. As this is a Physiotherapy intervention, which will randomise participants at the beginning of each intervention block there will be no need for out-of-hours randomisation procedures.

Allocation will be concealed in consecutively numbered opaque envelopes which will be drawn up for each block and presented to the treating physiotherapist after the baseline assessment session has been completed. Allocation will not be revealed until after the baseline assessment has been completed, and once revealed will be entered onto the enrolment log.

Blinding

For peer review only - http://bmjopen.bmj.com/site/about/guidelines.xhtml

This is a single (assessor) blinded proof of concept study. As this study is comparing a technology intervention (HOLOBalance) vs standard care it is not possible to blind the participants or the treating clinicians to the intervention provided. The blinded outcome assessor will collect all of the outcome measures at baseline and follow up. The outcome assessor will be asked to record any incidences of unblinding and detail how this occurred. Due to the nature of this study, we do not foresee circumstances where emergency unblinding of the outcome assessor is required.

Active Intervention: The HOLOBalance System

HOLOBalance is an augmented reality balance training programme based upon evidence-based multi-sensory rehabilitation (MSR) protocols [45, 46]. MSR requires individuals to regularly perform exercises which challenge the balance system (e.g. closed eyes, stand on foam), optimise inner ear balance function and have been shown to improve balance control in healthy older adults [45, 46] and people with inner ear balance dysfunction [47-50].

Participants will be required to wear a series of body worn sensors (pressure detecting insoles, accelerometer and a heart rate monitor, Figure 2) when performing the exercises so that 1) the computer software can assess the participants performance and 2) the user can interact with the system. HOLOBalance will provide prescribed exercises and exergames presented into the person's home environment by ahead mounted augmented reality display and log the user's interactions with the system (e.g. number of sessions completed). HOLOBalance will display a holographic virtual physiotherapist (Figure 3A) to provide instructions and demonstrate prescribed exercises. The HOLOBalance programme will include exercises to maximise opportunities for balance improvement and include turning the head whilst looking at a target, standing on foam, and walking whilst turning the head (for examples of gameplay please see figure 3B and 3C, and for table of basic exercises please see Table 1). In addition, participants will be given auditory and cognitive exercises in supplement to their balance training programme. For further information on the HOLOBalance intervention, please visit www.holobalance.eu.

For peer review only - http://bmjopen.bmj.com/site/about/guidelines.xhtml

Control Intervention: The OTAGO Home Exercise Programme

The OTAGO HEP booklet will be provided to all individuals in the control arm. Following the physiotherapy home assessment, participants will be provided with a list of exercises from the OTAGO booklet for them to complete every day. Participants will be required to log their exercises in an exercise diary for review by the treating clinician at the next home visit. Exercises will be progressed in line with the recommendations of the OTAGO HEP in combination with the treating clinician's clinical judgement.

Outcome Measures

Primary Outcomes

The primary outcomes will relate to the acceptability, safety and feasibility of providing the HOLOBalance intervention. Acceptability measures include; recruitment rate (percentage of eligible participants enrolled), compliance with interventions (percentage of prescribed sessions completed in HOLOBalance and control groups) and drop-out rates (number of drops outs in HOLOBalance and control groups). Additionally, exit interviews will be completed with participants that receive the HOLOBalance intervention.

In the HOLOBalance group, adherence will automatically be monitored through the interaction with the system, and calculated as the number of prescribed balance exercises, auditory and cognitive training tasks completed per day, expressed as a percentage. In the control group participants will be required to complete a daily exercise diary to report on their level of activity, whether they had completed their Home Exercise Programme session and whether they had performed their cognitive tasks. All participants will receive a weekly telephone call from the research physiotherapist to discuss any issues, their current progress and to remind them to complete their exercise diaries (if in the control group) which will be collected at their final outcome measure test session. The order of events for participants enrolled in this study is displayed in Table 2.

Safety of the HOLOBalance intervention will be assessed by recording and assessing all adverse / serious adverse events and device effects (and reporting these to appropriate agencies as required). The feasibility of the study will be explored by collecting information on and analysing any deviations from protocol or problems with implementing the study protocol (e.g. logistical problems).

Secondary outcomes

For peer review only - http://bmjopen.bmj.com/site/about/guidelines.xhtml

Balance Assessment

The mini BESTest [51] is a 14-item test that assesses dynamic balance components including anticipatory postural adjustments, reactive postural control, sensory orientation and dynamic gait. The data is based on a total score of 28 points. The test takes approximately 10 minutes to complete. This will be collected at baseline (week 0) and follow up (week 9)

The Functional Gait Assessment (FGA) [52] is a 10-item test that assesses performance on complex gait tasks (e.g. walking with head turns or stopping and turning). The test takes approximately 5 minutes to complete. This will be collected at baseline (week 0) and follow up (week 9)

Falls diaries will be provided for participants to record whether they have fallen each day. Participants will be required to return fall diaries in postage paid envelopes each month to the research team. New diaries will be sent at the end of each month to participants with a reminder for them to continue to complete diaries and send to the research team. Falls diaries will be collected for the duration of the intervention and for 6 months after completion of the intervention. If diaries are not received after one week of the start of the month, researchers will telephone the participant to ask whether it has been sent.

Cognitive Assessment

The validated Montreal Cognitive Assessment (MoCA) [53]: This test includes sections on visuospatial/executive function (alternating trail-making, cube copy, clock drawing), naming (lion, rhinoceros, camel), attention (forward and backward digit span, tapping to the letter A, subtracting 7s from 100), language (sentence repetition, letter fluency), abstraction (similarities between train and bicycle, watch and ruler), memory (delayed verbal recall of 5 words) and orientation to time and place (6 questions). This test will be used as a screening test for cognitive function prior to inclusion in the trial. This will be collected at baseline (week 0) and follow up (week 9)

The validated Cambridge Neuropsychological Test Automated Battery [54]; (CANTAB) cognitive test battery will be used to assess specific cognitive function domains pre-post intervention. The test is validated for use in people with MCI169 and has a specific test battery to assess for MCI. The test battery includes: (i) Motor screening task to assess a participant's general ability to understand and complete tasks and highlights if any sensorimotor or hearing impairments will have an impact on test performance, (ii) Paired Associated Learning assesses visual associative learning and memory, (iii) Spatial Working Memory to assess one's ability to retain and use visuospatial input, (iv) Reaction Time tests

For peer review only - http://bmjopen.bmj.com/site/about/guidelines.xhtml

a person's mental and motor response speed, (v) Rapid Visual Information Processing assesses the ability to maintain visual attention and continuous performance on a task and (vi) Delayed Matching to Sample tests visual recognition memory and short term visual memory. This assessment will take approximately 35 minutes to complete. Processing speed and executive functions from the CANTAB suite (http://www.cambridgecognition.com/academic/cantabsuite/battery) will be compiled to derive an executive function score. This will be collected at baseline (week 0) and follow up (week 9)

Physical Activity and Social Participation Assessment

The Rapid Assessment of Physical Activity [55] (RAPA) is a 9-item, self-administered questionnaire developed to provide an easily administered and interpreted means of assessing levels of physical activity among adults older than 50 years. RAPA evaluates a wide range of physical activity level, from sedentary to vigorous activity, as well as strength and flexibility training and takes 5-minutes to complete. It has adequate convergent validity and good criterion validity. This will be collected at baseline (week 0) and follow up (week8)

The World Health Organization Disability Assessment Schedule 2.0 [56] (WHODAS 2.0) is an assessment which provides a global measure of disability. It covers the following domains of functioning: Cognition – understanding & communicating; Mobility– moving & getting around; Self-care– hygiene, dressing, eating & staying alone; Getting along– interacting with other people; Life activities– domestic responsibilities, leisure, work & school; Participation– joining in community activities. This will be collected at baseline (week 0) and follow up (week 9)

Subjective Questionnaires

The Activities-specific Balance Confidence Scale [57] (ABC) assesses patient's perceived confidence for performing 16-activities of daily living without losing balance. Scores ≤67/100% indicate increased falls risk. This will be collected at baseline (week 0) and follow up (week 9)

The Falls Efficacy Scale International (FES-I) is a short, easy to administer tool measuring an individual's level of concern regarding falling during social and physical activities inside and outside the home, whether or not the person actually does the activity. Level of concern

For peer review only - http://bmjopen.bmj.com/site/about/guidelines.xhtml

For peer review only

is measured on a four-point Likert scale (1=not at all to 4=very). It has excellent internal validity and test-retest reliability. Scores of >23 for the long form and >10 for the short form have been suggested as cut points for indicating high concern about falling [58]. This will be collected at baseline (week 0) and follow up (week 9)

The EQ-5D [59] is a standardized, valid and reliable simple, generic measure of health status for clinical and economic appraisal. The EQ-5D-5L has 5 dimensions (mobility, selfcare, usual activities, pain/discomfort, anxiety/depression) and includes the EQ visual Analogue scale (EQ VAS). The respondent is asked to rate their health status on these five dimensions from 1 to 5 respectively as no problems, slight problems, moderate problems, severe problems, and extreme problems. The EQ VAS records the respondent's self-rated health on a 20 cm vertical, visual analogue scale with endpoints labelled 'the best health you can imagine' and 'the worst health you can imagine'. The respondent is asked to mark an X on the scale to indicate "how your health is TODAY". This will be collected at baseline (week 0) and follow up (week 9)

Environmental Analysis of Mobility [60] (EMS) scale is a self-report scale assessing the effect of the physical environment on community mobility. Twenty-four features of the physical environment are identified. For each feature, an encounter question (How often do you?) is paired with an avoidance question (How often do you avoid?). Subjects report on frequency of encounter and avoidance behaviour using a five-point ordinal scale (never, rarely, sometimes, often, and always). The test-retest reliability of the questionnaire is good. This will be collected at baseline (week 0) and follow up (week 9)

The behavioural regulation in exercise questionnaire (BREQ-3) [61, 62] is a 24 item questionnaire to assess motivation to exercise. Participants rate whether statements apply to themselves (or not) using a 5 point Likert scale ranging from 0 (Not true for me) to 4 (Very true for me). This will be collected at baseline (week 0) and follow up (week 9)

User Experience Evaluation (HOLOBalance Group Only)

The system usability scale (SUS) [63] is a 10 item questionnaire that asks individuals to rate their experience of using the system on a 5 point Likert scale ranging from 1 (strongly disagree) to 5 (strongly agree). Example items include 'I think that I wold like to use this product regularly'. This will be collected by the treating physiotherapist at week 6 only

The user experience questionnaire (UEQ) [64] is a 26 item self-report questionnaire to assess a person's experience of using a product. Participants rate the product between two

For peer review only - http://bmjopen.bmj.com/site/about/guidelines.xhtml

contrasting attributes (e.g. attractive vs unattractive) on a 7 point Likert scale. This will be collected by the treating physiotherapist at weeks 1,3 and 6.

The NASA Task Load Index (NASA-TLX)[65] is a widely used, subjective, multidimensional assessment tool that rates perceived workload in order to assess a task, system, or team's effectiveness or other aspects of performance. The NASA TLX assesses work load on five 21 point visual analogue scales ranging from 0 (Very Low) to 21 (Very high) for task demands including mental demand, physical demand and temporal demand. Questions from the NASA TLX will be integrated into the HOLOBalance tasks. These questions will be administered 20 times per participant in total

For peer review only

Data management and statistical analysis

Anonymised data will be inputted into a study database. The integrity of this data will be assessed by taking a random sample (10%) from the study database (after the completion of data collection) and comparing with the paper CRF. If there is less than 98% agreement between the paper CRF record and inputted measures, all data will be checked and rectified. The rectified database will be saved under a new filename (e.g. studydatabase_rectified_date) and all changes made to the database will be logged. Essential trial documentation will be kept with the Trial Master File and Investigator Site Files. The sponsor will ensure that study documentation is retained in accordance with their local approvals after the conclusion of the trial.

As this is a proof of concept study, there is no formal plan for statistical analysis due to the small sample size and lack of statistical power. However, for a future trial an analysis plan has been developed which will be run in this study to determine its appropriateness. We will use intention to treat analysis with repeated measures general linear model analysis of variance (ANOVA) for selected outcome measures and other appropriate tests for between-group and within group analysis. We will also include correlations analysis and chi-square assumption testing. Sex and gender analysis will be completed considering the ethical and equity impacts assessment, approaching, with a qualitative and a quantitative research tool, both the determinants of accessibility to the technology, and the determinants of social and health care costs in the two populations of potential users. We will enrol 80 participants altogether in order to be able to stratify results based on degree of balance impairment and cognitive function at baseline and gender. A considerable effect size of >1.35 is anticipated

For peer review only - http://bmjopen.bmj.com/site/about/guidelines.xhtml

as per our previous study on older adults [1]. After completion of the proof of concept study, the results will be collected and analysed in order to deliver the final validation report of the HOLOBALANCE platform.

Sample Size

This proof of concept study will recruit 60 participants per group (i.e. total sample size of 120). This will allow the researchers to gain preliminary data on the primary outcomes of safety, feasibility and acceptability of the HOLOBalance intervention. This will also provide sufficient data to explore trends for effectiveness and to allow for sample size estimates to be drawn up for a future trial.

Study Oversight

Trial steering committees (TSC) and Trial Management Groups (TMG) will be formed to oversee the conduct of the research according to predetermined terms of reference.

Patient Participant Involvement

As part of the larger HOLOBalance project, the research consortium has performed extensive PPI with older adults, discussing the study design, exergames and the supporting technologies with approximately 75 older adults in total.

ETHICS AND DISSEMINATION

This study has received institutional ethical approvals in Germany (reference: 265/19) and Greece (reference: 9769/24-6-2019) and from the Health Research Authority in the UK (reference: 19/LO/1908). All amendments to the study protocol which may impact on the integrity of the study, or the data are required to receive approval by the REC prior to their implementation.

Findings from this study will be provided as lay reports to participants, disseminated to community groups via community partners and will be submitted for peer reviewed publications. Additionally, electronic data will be anonymised and uploaded to a data repository (Zenodo) that supports restricted access. Electronic data will not be made publicly available and sharing will be made possible only by the approval of the Holobalance data

For peer review only - http://bmjopen.bmj.com/site/about/guidelines.xhtml

management board, and use and re-use of the pilot dataset will be subject to the license under which the data objects were deposited.

TRIAL REGISTRATION

This study has been prospectively registered on www.clinicaltrials.gov (identifier: NCT04053829).

TRIAL STATUS

This study is due to start recruiting April 2020

FUNDING STATEMENT

This project has received funding from the European Union's Horizon 2020 research and innovation programme under grant agreement No 769574. This article reflects only the author's view and the Commission is not responsible for any use that may be made of the information it contains

AUTHOR CONTRIBUTIONS

MBL, GG, DEB, MP were involved in the conception, design and writing and editing of the study protocol. DG, DF, DK and CM were involved in the conception and editing of the protocol. All authors approved the final protocol.

COMPETING INTERESTS

None

For peer review only - http://bmjopen.bmj.com/site/about/guidelines.xhtml

TABLES

Position	Description	Progressions	Progression Activation
**Seated**	Head turns side to side (yaw rotation of 30 degrees) whilst visually fixating on a static target placed at eye level in front of the participant	Increase speed of movements<br><br>Visual target moves in opposite direction to head movement	Increase speed of head turns if patient reporting no symptoms<br><br>If patient can perform 3 head turns (i.e. left to right) in one second without provoking symptoms, move to next progression
**Seated**	Had turns up and down (pitch rotation of 30 degrees) whilst visually fixating on a static target placed at eye level in front of the participant	Increase speed of movements<br><br>Visual target moves in opposite direction to head movement<br><br>Look up to ceiling and down to floor without fixation	Increase speed of head turns if patient reporting no symptoms<br><br>If patient can perform 3 head turns (i.e. left to right) in one second without provoking symptoms, move to next progression

For peer review only

**Seated**	Bend down to pick object up from the floor in front of you	Close the eyes<br><br>Reach to pick up objects to the side	If patient can complete the full range of movement consistently in 2 seconds (or less) in eyes open condition, and no symptoms are provoked. Progress to eyes closed.<br><br>When able to complete forward bends with eyes open and closed, progress to sideways bend.
**Standing**	Stand with feet hip width apart, looking straight ahead with eyes open	Bring feet closer together<br><br>Close the eyes	If patient can maintain stability in the correct position for one minute with eyes open without prompts, then ask patient to close eyes.<br><br>If patient can maintain stability in the correct position with eyes closed, reduce base of support.

For peer review only - http://bmjopen.bmj.com/site/about/guidelines.xhtml

**Standing**	Stand on foam cushion with feet hip width apart and looking straight ahead with eyes open	Bring feet closer together<br><br>Close the eyes	If patient can maintain stability in the correct position for one minute with eyes open without prompts, then ask patient to close eyes.<br><br>If patient can maintain stability in the correct position with eyes closed, reduce base of support.
**Standing**	Stand with feet hip width apart and bend over to pick up an object from the floor	Bring feet closer together<br><br>Reach up as if to reach into a cupboard	When patient can safely complete full movement in less than 3 seconds, and this produces no symptoms, ask patient to increase speed.<br><br>When patient can safely complete the full movement (without symptoms) in under 2 seconds, move on to the next exercise.

For peer review only - http://bmjopen.bmj.com/site/about/guidelines.xhtml

**Standing**	Step turn through 180° to face the opposite direction	Increase speed of movement	Once patient is able to perform the exercise safely and confidently at their baseline speed, ask them to increase their speed.
**Walking**	Walk forwards looking straight ahead	Increase walking speed	Once patient is able to perform the exercise safely and confidently at their baseline speed with no deviation, ask them to increase their gait speed.
**Walking**	Walk forwards across the room looking between two targets placed at eye level, 1.5 metres apart on the horizontal plane.	Increase walking speed<br><br>Increase head turn speed<br><br>Increase head turn amplitude when visual targets are removed	Once patient is able to perform the exercise safely and confidently at their baseline speed with no symptoms or deviation, ask them to increase their gait speed.<br><br>Once patient is able to perform the exercise safely and confidently with no symptoms or

For peer review only - http://bmjopen.bmj.com/site/about/guidelines.xhtml

			deviation, ask them to increase their head turn speed<br><br>Once patient is able to perform the exercise safely and confidently with no symptoms or deviation, ask them to increase amplitude of head turns
**Walking**	Walk across the room and nod your head to look up to the ceiling and down to the ground.	Increase walking speed<br><br>Integrate diagonal head movements	Once patient is able to perform the exercise safely and confidently at their baseline speed with no symptoms or deviation, ask them to increase their gait speed.<br><br>Once patient is able to perform the exercise safely and confidently with no symptoms or deviation, ask them to increase their head turn speed<br><br>Once patient is able to perform the exercise safely and confidently with no symptoms or deviation, ask them to integrate diagonal head movements

Table 1: The HOLOBalance intervention basic exercises and their progressions

For peer review only - http://bmjopen.bmj.com/site/about/guidelines.xhtml

		Screening Session	Baseline Test Session	Week 0	Daily	Weekly	Monthly	Week 3	Week 6	Follow-Up Test Session	Week 9
**Recruitment Procedures**	Provide Patient information Sheet	X									
	Provide informed consent		X								
**University-based data collection**	Demographics		X								
	The MOCA		X							X	
	The RAPA		X							X	
	The WHODAS 2.0		X							X	
	The CANTAB		X							X	
	The ABC Scale		X							X	
	The FES-I		X							X	
	The EQ5D		X							X	
	The EMA		X							X	
	The BREQ -3		X							X	
	Mini-BESTest		X							X	
	The FGA		X							X	
	Exit Interviews (HOLOBalance Group Only)										X
**Usability Measures**	The SUS								X		
	The UEQ			X				X	X		

For peer review only

**Home-based interactions**	Equipment installation & Removal (HOLOBalance Only)	X						X
	Home visit from Physiotherapist	X				X	X	
	Telephone conversation with physiotherapist			X				
	Perform Home Exercise Programme		X					
	Submit falls diaries				X			

For peer review only

Table 2: Order of events for all participants enrolled in the HOLOBalance study.

For peer review only - http://bmjopen.bmj.com/site/about/guidelines.xhtml

REFERENCES

1. Bridenbaugh, S.A. and R.W. Kressig, *Motor cognitive dual tasking.* 2015. **48**(1): p. 15-21.
2. Frewen, J., et al., *Recent syncope and unexplained falls are associated with poor cognitive performance.* Age Ageing, 2015. **44**(2): p. 282-286.
3. Horak, F.B., *Postural orientation and equilibrium: what do we need to know about neural control of balance to prevent falls?* Age Ageing, 2006. **35 Suppl 2**: p. ii7-ii11.
4. Agrawal, Y., et al., *Disorders of balance and vestibular function in US adults: data from the National Health and Nutrition Examination Survey, 2001-2004.* Arch Intern Med, 2009. **169**(10): p. 938-44.
5. Lord, S.R. and R.D. Clark, *Simple physiological and clinical tests for the accurate prediction of falling in older people.* Gerontology, 1996. **42**(4): p. 199-203.
6. Jacobson, G.P., et al., *Significant vestibular system impairment is common in a cohort of elderly patients referred for assessment of falls risk.* J Am Acad Audiol, 2008. **19**(10): p. 799-807.
7. Liston, M.B., et al., *Peripheral vestibular dysfunction is prevalent in older adults experiencing multiple non-syncopal falls versus age-matched non-fallers: a pilot study.* Age Ageing, 2014. **43**(1): p. 38-43.
8. Agrawal, Y., *Editorial: Age-Related Vestibular Loss: Current Understanding and Future Research Directions.* Front Neurol, 2017. **8**: p. 443.
9. Baldursdottir, B., et al., *Sensory impairments and wrist fractures: A case-control study.* J Rehabil Med, 2018. **50**(2): p. 209-215.
10. Kristinsdottir, E.K., G.B. Jarnlo, and M. Magnusson, *Asymmetric vestibular function in the elderly might be a significant contributor to hip fractures.* Scand J Rehabil Med, 2000. **32**(2): p. 56-60.
11. Kristinsdottir, E.K., et al., *Observation of vestibular asymmetry in a majority of patients over 50 years with fall-related wrist fractures.* Acta Otolaryngol, 2001. **121**(4): p. 481-5.
12. Ble, A., et al., *Executive function correlates with walking speed in older persons: the InCHIANTI study.* J Am Geriatr Soc, 2005. **53**(3): p. 410-5.
13. Coppin, A.K., et al., *Association of executive function and performance of dual-task physical tests among older adults: analyses from the InChianti study.* Age Ageing, 2006. **35**(6): p. 619-24.
14. Kearney, F.C., et al., *The relationship between executive function and falls and gait abnormalities in older adults: a systematic review.* Dement Geriatr Cogn Disord, 2013. **36**(1-2): p. 20-35.
15. Liu-Ambrose, T., et al., *Dual-task gait performance among community-dwelling senior women: the role of balance confidence and executive functions.* J Gerontol A Biol Sci Med Sci, 2009. **64**(9): p. 975-82.
16. Rossi, A.F., et al., *The prefrontal cortex and the executive control of attention.* Experimental brain research. Experimentelle Hirnforschung. Experimentation cerebrale, 2009. **192**(3): p. 489-497.
17. Vaughan, L. and K. Giovanello, *Executive function in daily life: Age-related influences of executive processes on instrumental activities of daily living.* Psychol Aging, 2010. **25**(2): p. 343-55.
18. Yogev-Seligmann, G., J.M. Hausdorff, and N. Giladi, *The role of executive function and attention in gait.* Mov Disord, 2008. **23**(3): p. 329-42; quiz 472.
19. Boyle, P.A., et al., *Executive dysfunction and apathy predict functional impairment in Alzheimer disease.* Am J Geriatr Psychiatry, 2003. **11**(2): p. 214-21.

For peer review only - http://bmjopen.bmj.com/site/about/guidelines.xhtml

20. Muir, S.W., K. Gopaul, and M.M. Montero Odasso, *The role of cognitive impairment in fall risk among older adults: a systematic review and meta-analysis.* 2012. **41**(3): p. 299-308.
21. Delbaere, K., et al., *Mild cognitive impairment as a predictor of falls in community-dwelling older people.* Am J Geriatr Psychiatry, 2012. **20**(10): p. 845-53.
22. Dommes, A. and V. Cavallo, *The role of perceptual, cognitive, and motor abilities in street-crossing decisions of young and older pedestrians.* Ophthalmic Physiol Opt, 2011. **31**(3): p. 292-301.
23. World Health Organization. *Physical Activity*. 2018; Available from: https://www.who.int/news-room/fact-sheets/detail/physical-activity.
24. Public Health England, *Falls and Fracture consensus statement*. 2017, Public Health England.
25. Public Health England. *Physical Activity: applying All Our Health*. 2019 20-12-2019]; Available from: https://www.gov.uk/government/publications/physical-activity-applying-all-our-health/physical-activity-applying-all-our-health.
26. National Institute for Heath and Care Excellence, *Falls in Older People: Assessing Risk and Prevention (CG161).* 2013, NICE: UK.
27. Physicians, R.C.o., *Falling standards broken promises: Report of the national audit of falls and bone health in older people 2010*. 2011, Royal College of Physicians.
28. Sherrington, C., et al., *Exercise to prevent falls in older adults: an updated systematic review and meta-analysis.* Br J Sports Med, 2017. **51**(24): p. 1750-1758.
29. Hall, C.D., et al., *Vestibular Rehabilitation for Peripheral Vestibular Hypofunction: An Evidence-Based Clinical Practice Guideline: FROM THE AMERICAN PHYSICAL THERAPY ASSOCIATION NEUROLOGY SECTION.* J Neurol Phys Ther, 2016. **40**(2): p. 124-55.
30. Sherrington, C., et al., *Exercise for preventing falls in older people living in the community: an abridged Cochrane systematic Review.* British Journal of Sports Medicine, 2019: p. bjsports-2019-1.
31. Pavlou, M., A.M. Bronstein, and R.A. Davies, *Randomized trial of supervised versus unsupervised optokinetic exercise in persons with peripheral vestibular disorders.* Neurorehabil Neural Repair, 2013. **27**(3): p. 208-18.
32. Campbell, A.J., et al., *Falls prevention over 2 years: a randomized controlled trial in women 80 years and older.* Age Ageing, 1999. **28**(6): p. 513-8.
33. Robertson, M.C., et al., *Preventing injuries in older people by preventing falls: a meta-analysis of individual-level data.* J Am Geriatr Soc, 2002. **50**(5): p. 905-11.
34. Robertson, M.C., et al., *Effectiveness and economic evaluation of a nurse delivered home exercise programme to prevent falls. 1: Randomised controlled trial.* BMJ, 2001. **322**(7288): p. 697-701.
35. Robertson, M.C., et al., *Effectiveness and economic evaluation of a nurse delivered home exercise programme to prevent falls. 2: Controlled trial in multiple centres.* BMJ, 2001. **322**(7288): p. 701-4.
36. Skelton, D. and S. Dinan, *Exercise for falls management: Rationale for an exercise programme aimed at reducing postural instability.* Physiotherapy Theory and Practise, 1999. **15**: p. 105-20.
37. Skelton, D., et al., *Tailored group exercise (Falls Management Exercise -- FaME) reduces falls in community-dwelling older frequent fallers (an RCT).* Age Ageing, 2005. **34**(6): p. 636-9.
38. Falbo, S., et al., *Effects of Physical-Cognitive Dual Task Training on Executive Function and Gait Performance in Older Adults: A Randomized Controlled Trial.* BioMed Research International, 2016. **2016**: p. 1-12.
39. Silsupadol, P., et al., *Training-related changes in dual-task walking performance of elderly persons with balance impairment: a double-blind, randomized controlled trial.* Gait Posture, 2009. **29**(4): p. 634-9.

For peer review only

40. Wollesen, B., et al., *Effects of Dual-Task Management and Resistance Training on Gait Performance in Older Individuals: A Randomized Controlled Trial.* Frontiers in Aging Neuroscience, 2017. **9**.
41. Liston, M.B., et al., *Feasibility and effect of supplementing a modified OTAGO intervention with multisensory balance exercises in older people who fall: a pilot randomized controlled trial.* Clin Rehabil, 2014. **28**(8): p. 784-793.
42. Baldursdottir, B., et al., *Multi-sensory training and wrist fractures: a randomized, controlled trial.* Aging Clinical and Experimental Research, 2019.
43. Kristinsdottir, E.K. and B. Baldursdottir, *Effect of multi-sensory balance training for unsteady elderly people: pilot study of the "Reykjavik model".* Disability and rehabilitation, 2014. **36**(14): p. 1211-1218.
44. Chan, A.-W., et al., *SPIRIT 2013 Statement: Defining Standard Protocol Items for Clinical Trials.* Annals of Internal Medicine, 2013. **158**(3): p. 200.
45. Hu, M.H. and M.H. Woollacott, *Multisensory training of standing balance in older adults: II. Kinematic and electromyographic postural responses.* J Gerontol, 1994. **49**(2): p. M62-71.
46. Hu, M.H. and M.H. Woollacott, *Multisensory training of standing balance in older adults: I. Postural stability and one-leg stance balance.* J Gerontol, 1994. **49**(2): p. M52-61.
47. Shumway-Cook, A. and F.B. Horak, *Rehabilitation strategies for patients with vestibular deficits.* Neurol Clin, 1990. **8**(2): p. 441-57.
48. Whitney, S.L., A.A. Alghwiri, and A. Alghadir, *An overview of vestibular rehabilitation.* Handb Clin Neurol, 2016. **137**: p. 187-205.
49. Whitney, S.L. and P.J. Sparto, *Principles of vestibular physical therapy rehabilitation.* NeuroRehabilitation, 2011. **29**(2): p. 157-66.
50. Hall, C.D., et al., *Vestibular Rehabilitation for Peripheral Vestibular Hypofunction.* Journal of Neurologic Physical Therapy, 2016. **40**(2): p. 124-155.
51. Franchignoni, F., et al., *Using psychometric techniques to improve the Balance Evaluation Systems Test: the mini-BESTest.* J Rehabil Med, 2010. **42**(4): p. 323-31.
52. Wrisley, D.M. and N.A. Kumar, *Functional gait assessment: concurrent, discriminative, and predictive validity in community-dwelling older adults.* Phys Ther, 2010. **90**(5): p. 761-73.
53. Nasreddine, Z.S., et al., *The Montreal Cognitive Assessment, MoCA: a brief screening tool for mild cognitive impairment.* J Am Geriatr Soc, 2005. **53**(4): p. 695-9.
54. Smith, P.J., et al., *A comparison of the Cambridge Automated Neuropsychological Test Battery (CANTAB) with "traditional" neuropsychological testing instruments.* Journal of Clinical and Experimental Neuropsychology, 2013. **35**(3): p. 319-328.
55. Topolski, T.D., et al., *The Rapid Assessment of Physical Activity (RAPA) among older adults.* Preventing chronic disease, 2006. **3**(4): p. A118-A118.
56. Silveira, C., et al., *Validation of the WHO Disability Assessment Schedule (WHODAS 2.0) 12-item tool against the 36-item version for measuring functioning and disability associated with pregnancy and history of severe maternal morbidity.* International Journal of Gynecology & Obstetrics, 2018. **141**: p. 39-47.
57. Powell, L.E. and A.M. Myers, *The Activities-specific Balance Confidence (ABC) Scale.* J Gerontol A Biol Sci Med Sci, 1995. **50A**(1): p. M28-34.
58. Delbaere, K., et al., *The Falls Efficacy Scale International (FES-I). A comprehensive longitudinal validation study.* Age and Ageing, 2010. **39**(2): p. 210-216.
59. *EuroQol - a new facility for the measurement of health-related quality of life.* Health Policy, 1990. **16**(3): p. 199-208.
60. Shumway-Cook, A., et al., *Assessing Environmentally Determined Mobility Disability: Self-Report Versus Observed Community Mobility.* Journal of the American Geriatrics Society, 2005. **53**(4): p. 700-704.
61. Wilson, P.M., et al., *"It's Who I Am … Really!' The Importance of Integrated Regulation in Exercise Contexts1.* 2007. **11**(2): p. 79-104.

For peer review only - http://bmjopen.bmj.com/site/about/guidelines.xhtml

62. Markland, D. and V. Tobin, *A modification to the Behavioural Regulation in Exercise Questionnaire to include an assessment of amotivation.* Journal of Sport & Exercise Psychology, 2004. **26**(2): p. 191-196.
63. Lewis, J.R., *The System Usability Scale: Past, Present, and Future.* International Journal of Human–Computer Interaction, 2018. **34**(7): p. 577-590.
64. Laugwitz, B., T. Held, and M. Schrepp. *Construction and Evaluation of a User Experience Questionnaire*. in *HCI and Usability for Education and Work*. 2008. Berlin, Heidelberg: Springer Berlin Heidelberg.
65. Hart, S.G. and L.E. Staveland, *Development of NASA-TLX (Task Load Index): Results of Empirical and Theoretical Research*, in *Advances in Psychology*, P.A. Hancock and N. Meshkati, Editors. 1988, North-Holland. p. 139-183.

For peer review only

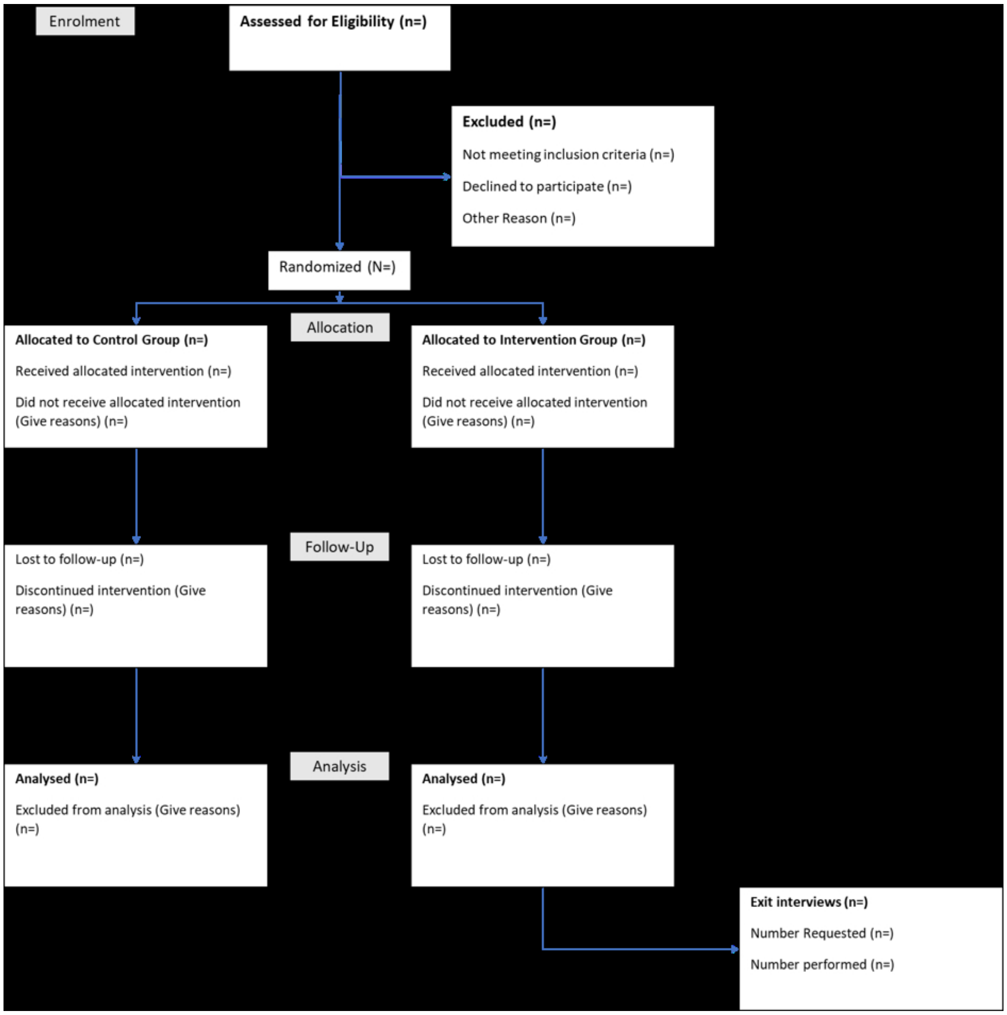

Consort Diagram for HOLOBalance Study

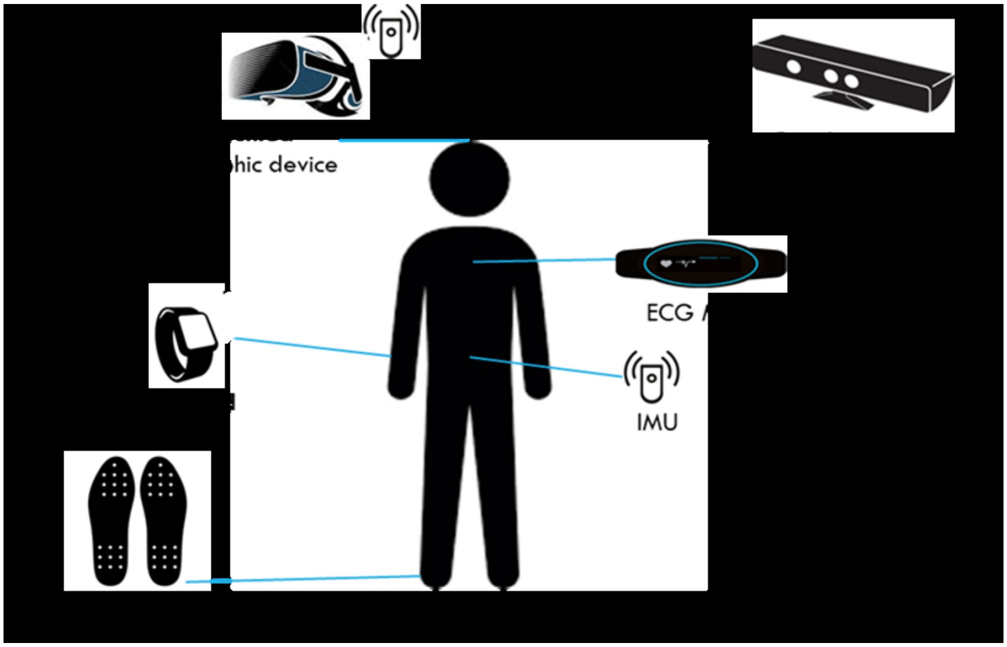

Schematic diagram of the HOLOBalance hardware

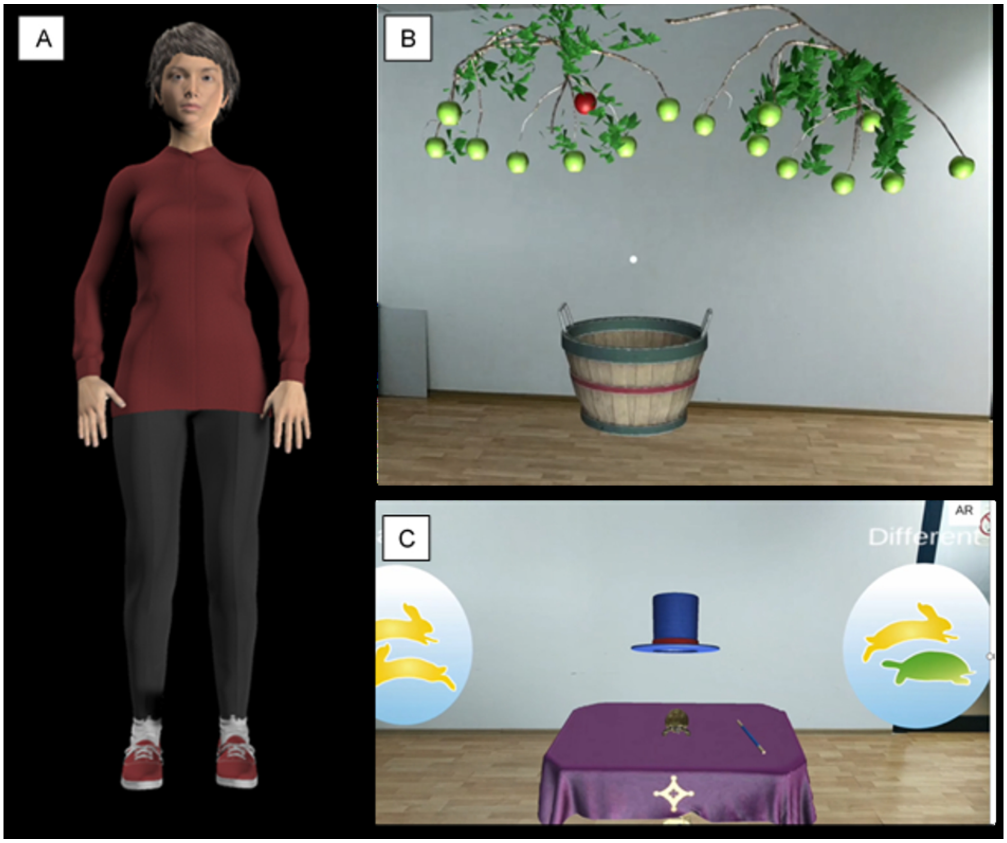

The hologram physiotherapist (panel A), and representative augmented reality gameplay from HOLOBalance (panels B and C).

Reporting checklist for protocol of a clinical trial.

Based on the SPIRIT guidelines.

Instructions to authors

Complete this checklist by entering the page numbers from your manuscript where readers will find each of the items listed below.

Your article may not currently address all the items on the checklist. Please modify your text to include the missing information. If you are certain that an item does not apply, please write "n/a" and provide a short explanation.

Upload your completed checklist as an extra file when you submit to a journal.

In your methods section, say that you used the SPIRITreporting guidelines, and cite them as:

Chan A-W, Tetzlaff JM, Altman DG, Laupacis A, Gøtzsche PC, Krleža-Jerić K, Hróbjartsson A, Mann H, Dickersin K, Berlin J, Doré C, Parulekar W, Summerskill W, Groves T, Schulz K, Sox H, Rockhold FW, Rennie D, Moher D. SPIRIT 2013 Statement: Defining standard protocol items for clinical trials. Ann Intern Med. 2013;158(3):200-207

		Reporting Item	Page Number
**Administrative information**			
Title	#1	Descriptive title identifying the study design, population, interventions, and, if applicable, trial acronym	1

For peer review only

Trial registration	#2a	Trial identifier and registry name. If not yet registered, name of intended registry	2 and 13
Trial registration: data set	#2b	All items from the World Health Organization Trial Registration Data Set	NA, This information is included in Clinical Trial Registration
Protocol version	#3	Date and version identifier	1
Funding	#4	Sources and types of financial, material, and other support	13
Roles and responsibilities: contributorship	#5a	Names, affiliations, and roles of protocol contributors	1
Roles and responsibilities: sponsor contact information	#5b	Name and contact information for the trial sponsor	13
Roles and responsibilities: sponsor and funder	#5c	Role of study sponsor and funders, if any, in study design; collection, management, analysis, and interpretation of data; writing of the report; and the decision to submit the report for publication, including whether they will have ultimate authority over any of these activities	13

For peer review only

Roles and responsibilities: committees	#5d	Composition, roles, and responsibilities of the coordinating centre, steering committee, endpoint adjudication committee, data management team, and other individuals or groups overseeing the trial, if applicable (see Item 21a for data monitoring committee)	13
**Introduction**			
Background and rationale	#6a	Description of research question and justification for undertaking the trial, including summary of relevant studies (published and unpublished) examining benefits and harms for each intervention	3
Background and rationale: choice of comparators	#6b	Explanation for choice of comparators	5
Objectives	#7	Specific objectives or hypotheses	8
Trial design	#8	Description of trial design including type of trial (eg, parallel group, crossover, factorial, single group), allocation ratio, and framework (eg, superiority, equivalence, non-inferiority, exploratory)	4
**Methods: Participants,**			

For peer review only

**interventions, and outcomes**			
Study setting	#9	Description of study settings (eg, community clinic, academic hospital) and list of countries where data will be collected. Reference to where list of study sites can be obtained	5
Eligibility criteria	#10	Inclusion and exclusion criteria for participants. If applicable, eligibility criteria for study centres and individuals who will perform the interventions (eg, surgeons, psychotherapists)	5,6
Interventions: description	#11a	Interventions for each group with sufficient detail to allow replication, including how and when they will be administered	7,8. Table 1.
Interventions: modifications	#11b	Criteria for discontinuing or modifying allocated interventions for a given trial participant (eg, drug dose change in response to harms, participant request, or improving / worsening disease)	8, Table 1
Interventions: adherance	#11c	Strategies to improve adherence to intervention protocols, and any procedures for monitoring adherence (eg, drug tablet return; laboratory tests)	7,8

For peer review only

Interventions: concomitant care	#11d	Relevant concomitant care and interventions that are permitted or prohibited during the trial	5
Outcomes	#12	Primary, secondary, and other outcomes, including the specific measurement variable (eg, systolic blood pressure), analysis metric (eg, change from baseline, final value, time to event), method of aggregation (eg, median, proportion), and time point for each outcome. Explanation of the clinical relevance of chosen efficacy and harm outcomes is strongly recommended	8-12
Participant timeline	#13	Time schedule of enrolment, interventions (including any run-ins and washouts), assessments, and visits for participants. A schematic diagram is highly recommended (see Figure)	Fig 1, Table 2
Sample size	#14	Estimated number of participants needed to achieve study objectives and how it was determined, including clinical and statistical assumptions supporting any sample size calculations	12
Recruitment	#15	Strategies for achieving adequate participant enrolment to reach target sample size	6

For peer review only

Methods: Assignment of interventions (for controlled trials)

Allocation: sequence generation	#16a	Method of generating the allocation sequence (eg, computer-generated random numbers), and list of any factors for stratification. To reduce predictability of a random sequence, details of any planned restriction (eg, blocking) should be provided in a separate document that is unavailable to those who enrol participants or assign interventions	6
Allocation concealment mechanism	#16b	Mechanism of implementing the allocation sequence (eg, central telephone; sequentially numbered, opaque, sealed envelopes), describing any steps to conceal the sequence until interventions are assigned	6
Allocation: implementation	#16c	Who will generate the allocation sequence, who will enrol participants, and who will assign participants to interventions	6
Blinding (masking)	#17a	Who will be blinded after assignment to interventions (eg, trial participants, care	7

For peer review only

		providers, outcome assessors, data analysts), and how	
Blinding (masking): emergency unblinding	#17b	If blinded, circumstances under which unblinding is permissible, and procedure for revealing a participant's allocated intervention during the trial	7
**Methods: Data collection, management, and analysis**			
Data collection plan	#18a	Plans for assessment and collection of outcome, baseline, and other trial data, including any related processes to promote data quality (eg, duplicate measurements, training of assessors) and a description of study instruments (eg, questionnaires, laboratory tests) along with their reliability and validity, if known. Reference to where data collection forms can be found, if not in the protocol	8-12
Data collection plan: retention	#18b	Plans to promote participant retention and complete follow-up, including list of any outcome data to be collected for participants who discontinue or deviate from intervention protocols	7-8

For peer review only

Data management	#19	Plans for data entry, coding, security, and storage, including any related processes to promote data quality (eg, double data entry; range checks for data values). Reference to where details of data management procedures can be found, if not in the protocol	12
Statistics: outcomes	#20a	Statistical methods for analysing primary and secondary outcomes. Reference to where other details of the statistical analysis plan can be found, if not in the protocol	12-13
Statistics: additional analyses	#20b	Methods for any additional analyses (eg, subgroup and adjusted analyses)	12
Statistics: analysis population and missing data	#20c	Definition of analysis population relating to protocol non-adherence (eg, as randomised analysis), and any statistical methods to handle missing data (eg, multiple imputation)	12
**Methods: Monitoring**			
Data monitoring: formal committee	#21a	Composition of data monitoring committee (DMC); summary of its role and reporting structure; statement of whether it is independent from the sponsor and competing interests; and reference to where further	NA. This was not deemed as necessary as this is a low risk study with short duration (data

For peer review only

		details about its charter can be found, if not in the protocol. Alternatively, an explanation of why a DMC is not needed	collection less than 12 months)
Data monitoring: interim analysis	#21b	Description of any interim analyses and stopping guidelines, including who will have access to these interim results and make the final decision to terminate the trial	8
Harms	#22	Plans for collecting, assessing, reporting, and managing solicited and spontaneously reported adverse events and other unintended effects of trial interventions or trial conduct	8
Auditing	#23	Frequency and procedures for auditing trial conduct, if any, and whether the process will be independent from investigators and the sponsor	8
**Ethics and dissemination**			
Research ethics approval	#24	Plans for seeking research ethics committee / institutional review board (REC / IRB) approval	13
Protocol amendments	#25	Plans for communicating important protocol modifications (eg, changes to eligibility criteria, outcomes, analyses) to relevant	13

For peer review only

		parties (eg, investigators, REC / IRBs, trial participants, trial registries, journals, regulators)	
Consent or assent	#26a	Who will obtain informed consent or assent from potential trial participants or authorised surrogates, and how (see Item 32)	6
Consent or assent: ancillary studies	#26b	Additional consent provisions for collection and use of participant data and biological specimens in ancillary studies, if applicable	Appendix 1 (CF)
Confidentiality	#27	How personal information about potential and enrolled participants will be collected, shared, and maintained in order to protect confidentiality before, during, and after the trial	12,13
Declaration of interests	#28	Financial and other competing interests for principal investigators for the overall trial and each study site	14
Data access	#29	Statement of who will have access to the final trial dataset, and disclosure of contractual agreements that limit such access for investigators	13
Ancillary and post trial care	#30	Provisions, if any, for ancillary and post-trial care, and for compensation to those who suffer harm from trial participation	Appendix 2 (PIS)

For peer review only

Dissemination policy: trial results	#31a	Plans for investigators and sponsor to communicate trial results to participants, healthcare professionals, the public, and other relevant groups (eg, via publication, reporting in results databases, or other data sharing arrangements), including any publication restrictions	13
Dissemination policy: authorship	#31b	Authorship eligibility guidelines and any intended use of professional writers	14
Dissemination policy: reproducible research	#31c	Plans, if any, for granting public access to the full protocol, participant-level dataset, and statistical code	13
**Appendices**			
Informed consent materials	#32	Model consent form and other related documentation given to participants and authorised surrogates	Appendix 1 and 2
Biological specimens	#33	Plans for collection, laboratory evaluation, and storage of biological specimens for genetic or molecular analysis in the current trial and for future use in ancillary studies, if applicable	Appendix 1 and 2

None The SPIRIT checklist is distributed under the terms of the Creative Commons Attribution License CC-BY-ND 3.0. This checklist can be completed online using https://www.goodreports.org/, a tool made by the EQUATOR Network in collaboration with Penelope.ai

For peer review only - http://bmjopen.bmj.com/site/about/guidelines.xhtml

BMJ Open

Investigating the Feasibility and Acceptability of the HOLOBalance System Compared to Standard Care in Older Adults at Risk for Falls: Study protocol for an assessor blinded pilot randomised-controlled study.

Journal:	*BMJ Open*
Manuscript ID	bmjopen-2020-039254.R1
Article Type:	Protocol
Date Submitted by the Author:	29-Aug-2020
Complete List of Authors:	Liston, Matthew; King's College London, Centre for Human and Applied Physiological Sciences<br>Genna, Gregory; University College London,<br>Maurer, Christoph; University of Freiburg, Department of Neurology and Neuroscience, Medical Center,<br>Kikidis, Dimitris; National and Kapodistrian University of Athens<br>Gatsios, Dimitris; University of Ioannina, Unit of Medical Technology and Intelligent Information Systems, Department of Material Science and Engineering; University of Ioannina, Department of Neurology, Medical School,<br>Fotiadis, Dimitris; University of Ioannina, Unit of Medical Technology and Intelligent Information Systems, Department of Material Science and Engineering<br>Bamiou, Doris-Eva; UCL, The Ear Institute<br>Pavlou, Marousa; King's College London, Centre for Human and Applied Physiological Sciences
**Primary Subject Heading**:	Rehabilitation medicine
Secondary Subject Heading:	Geriatric medicine
Keywords:	GERIATRIC MEDICINE, REHABILITATION MEDICINE, Telemedicine < BIOTECHNOLOGY & BIOINFORMATICS

SCHOLARONE™ Manuscripts

I, the Submitting Author has the right to grant and does grant on behalf of all authors of the Work (as defined in the below author licence), an exclusive licence and/or a non-exclusive licence for contributions from authors who are: i) UK Crown employees; ii) where BMJ has agreed a CC-BY licence shall apply, and/or iii) in accordance with the terms applicable for US Federal Government officers or employees acting as part of their official duties; on a worldwide, perpetual, irrevocable, royalty-free basis to BMJ Publishing Group Ltd ("BMJ") its licensees and where the relevant Journal is co-owned by BMJ to the co-owners of the Journal, to publish the Work in this journal and any other BMJ products and to exploit all rights, as set out in our licence.

The Submitting Author accepts and understands that any supply made under these terms is made by BMJ to the Submitting Author unless you are acting as an employee on behalf of your employer or a postgraduate student of an affiliated institution which is paying any applicable article publishing charge ("APC") for Open Access articles. Where the Submitting Author wishes to make the Work available on an Open Access basis (and intends to pay the relevant APC), the terms of reuse of such Open Access shall be governed by a Creative Commons licence – details of these licences and which Creative Commons licence will apply to this Work are set out in our licence referred to above.

Other than as permitted in any relevant BMJ Author's Self Archiving Policies, I confirm this Work has not been accepted for publication elsewhere, is not being considered for publication elsewhere and does not duplicate material already published. I confirm all authors consent to publication of this Work and authorise the granting of this licence.

For peer review only

Investigating the Feasibility and Acceptability of the HOLOBalance System Compared to Standard Care in Older Adults at Risk for Falls: Study protocol for an assessor blinded pilot randomised-controlled study.

Matthew B Liston[1], Gregory J Genna[2], Christoph Maurer[3], Dimitris Kikidis[4], Dimitris Gatsios[5,6,] Dimitris Fotiadis, Doris Eva-Bamiou[2,7,8], Marousa Pavlou[1].

[1] Centre for Human and Applied Physiological Sciences, King's College London. UK
[2]The Ear Institute, University College London, London, UK
[3] Department of Neurology and Neuroscience, Medical Center, University of Freiburg, Freiburg, Germany
[4] Department of Otolaryngology, National and Kapodistrian University of Athens, Athens, Greece
[5]Department of Neurology, Medical School, University of Ioannina, Ioannina, Greece
[6]Unit of Medical Technology and Intelligent Information Systems, Department of Material Science and Engineering, University of Ioannina, Ioannina, Greece
[7] Neuro-Otology Department, University College London Hospitals, London, UK
[8]Biomedical Research Centre, National Institute for Health Research, London, UK

CORRESPONDING AUTHOR:

Liston, Matthew B. Centre for Human and Applied Physiological Sciences, King's College London. London, United Kingdom. SE1 1UL. Matthew.Liston@kcl.ac.uk

WORD COUNT

3914 words

PROTOCOL VERSION: Version 2, 20th January 2020

For peer review only - http://bmjopen.bmj.com/site/about/guidelines.xhtml

ABSTRACT

Introduction: Approximately 1/3 of all older adults fall each year, with wide ranging physical, psychosocial and healthcare related consequences. Exercise-based interventions are the cornerstone for falls prevention programmes, yet these are not consistently provided, do not routinely address all components of the balance system, and are often not well attended. The HOLOBalance system provides an evidence-based balance training programme delivered to patients in their home environment using a novel technological approach including an augmented reality virtual physiotherapist, exergames and a remote monitoring system. The aims of this proof of concept study are to 1) determine the safety, acceptability and feasibility of providing HOLOBalance to community dwelling older adults at risk for falls and 2) provide data to support sample size estimates for a future trial.

Methods: A single (assessor) blinded pilot randomised controlled proof of concept study. 120 participants will be randomised to receive an 8-week home exercise programme consisting of either: 1) HOLOBalance, or 2) The OTAGO Home Exercise Programme. Participants will be required to complete their exercise programme independently under the supervision of a physiotherapist. Participants will have weekly telephone contact with their physiotherapist, and will receive home visits at weeks 0, 3 and 6. Outcome measures of safety, acceptability and feasibility, clinical measures of balance function, disability, balance confidence and cognitive function will be assessed before and immediately after the 8 week intervention. Acceptability and feasibility will be explored using descriptive statistics, and trends for effectiveness will be explored using GLM ANOVA.

Ethics and Dissemination: This study has received institutional ethical approvals in Germany (reference: 265/19), Greece (reference: 9769/24-6-2019) and the UK (reference: 19/LO/1908). Findings from this study will be submitted for peer reviewed publications.

Trial Registration Number: NCT04053829.

Keywords: Older Adults, Falls Prevention, Falls Rehabilitation, Tele-rehabilitation, Protocol.

STRENGTHS AND LIMITATIONS OF THIS STUDY

This study utilises a pilot randomised control design to explore the safety, acceptability and feasibility of providing the HOLOBalance intervention compared to an established home exercise programme (the Otago HEP).

The HOLOBalance programme provides an individualised training programme prescribed by a treating clinician which addresses all facets of the postural control system.

For peer review only - http://bmjopen.bmj.com/site/about/guidelines.xhtml

The HOLOBalance study will use quantitative and qualitative data to explore the acceptability of providing holographic balance training programmes to older adults at risk for falls.

This study will provide crucial insight into the feasibility of implementing state-of-the-art technologies in the home environment.

This pilot study is not powered to determine whether HOLOBalance reduces falls risk or falls rate

For peer review only

For peer review only - http://bmjopen.bmj.com/site/about/guidelines.xhtml

INTRODUCTION

One in three people over the age of 65 fall annually [1]It is widely accepted that falls are multi-factorial in nature [2, 3] and that impaired balance function is associated with falls [4]. Maintaining balance is complex and depends upon sensory inputs from the visual, proprioceptive and vestibular systems to provide information on body position and motion. Alongside this, central processes monitor and control the interaction between musculoskeletal and neural systems to generate anticipatory postural adjustments and adapt posture to changing environmental and balance task demands [5]. Age-related decline in these systems, are well documented and lead to impaired postural and gait control thus increasing risk for falls [6, 7]. Recent studies have identified that vestibular dysfunction is common in older adults who fall [6, 8-13], with up to 80% of fallers experiencing vestibular dysfunction [9]. Although sensory inputs are crucial for postural control, cognitive functions are equally as important for maintaining safe community ambulation. Deficits in executive function and attention are common in older age and are associated with impairments in postural control, dual-tasking balance ability (i.e. walk and talk at the same time), reduced gait speed and increased falls risk [14-20]. Additionally, changes in cognitive function strongly predict impairment in activities of daily living and functional independence [19, 21-23]. Thus, falls must be considered as multi-factorial in nature and should be targeted in interventions that address domains that impact upon balance and quality of life.

As falls have wide-ranging physical and psychological consequences and increase the likelihood of frailty, cognitive decline, sedentary behaviour, social exclusion, injury and death [5-7, 21, 24-26], their prevention is of primary concern to many public health bodies [27-29]. The National Institute of Clinical Excellence (NICE) UK Guidelines [30] recommend that older adults at risk for falls should undergo a balance assessment and receive targeted interventions to improve balance and reduce risk for falls. Despite the strength of available evidence, compliance and implementation for providing balance rehabilitation has been poor in UK healthcare settings [31].

Balance rehabilitation typically consists of personalized sets of exercises, defined by a healthcare professional and is the only effective treatment for balance disorders, irrespective of a person's age [32-34]. Although exercises are typically brief and easy to perform, there is up to 50% loss in follow up rate in older adults in these programmes, and while supervision significantly increases compliance and effectiveness [35], it is costly to provide. To this end, many falls prevention programmes provide exercise programmes completed either in group settings or independently at home [36-41]. These programmes have been shown to be effective in reducing falls rates in older adults, reducing falls by 30-40% (dependant on target

For peer review only - http://bmjopen.bmj.com/site/about/guidelines.xhtml

population), however they are limited in that they either do not provide exercises to address vestibular function or dual task training [37-39], or these are not introduced until very late in the programme [40, 41]. This is despite promising evidence suggesting that combined cognitive and functional training may provide improvements beyond single task training [42-44]. Similarly, rehabilitation programmes which address vestibular dysfunction (i.e. multi-sensory rehabilitation programmes) have been developed for older adults at risk for falls and have shown substantial additional reduction of falls risk to standard programmes [45-47]. These programmes require customised, expert led interventions to optimise recovery, however there is limited availability of experts to provide these individualised interventions and these may hinder their translation into clinical practice.

The HOLOBalance tele-rehabilitation platform has been developed to address this lack of expert physiotherapists. HOLOBalance will provide a customised and interactive falls rehabilitation programme that incorporates: 1) functional balance training, 2) multi-sensory exercises to improve balance function and 3) cognitive-motor training, for older adults at risk for falls. HOLOBalance will use off-the-shelf technologies to allow clinicians to provide an individualised balance rehabilitation programme for older adults who will be able to participate in at a time of their choice and in their home environment (for system description please visit www.holobalance.eu). This study protocol outlines the multi-site randomised controlled proof of concept study to explore safety, feasibility and acceptability of the HOLOBalance tele-rehabilitation system.

For peer review only

METHODS AND ANALYSIS

Trial Design

This study is an assessor-blinded, randomised proof of concept study to explore the acceptability and feasibility of providing a home-based balance tele-rehabilitation programme (HOLOBalance) to community dwelling older adults at risk for falls. All participants will be enrolled in a 8-week home exercise programme designed to improve balance function, and which adheres to the current guidance for strength and balance training [30]. All participants will receive an exercise programme provided under the supervision of a physiotherapist with regular reviews by telephone (weekly) and home visits (Week 0,3 and 6). Exercises will be

For peer review only - http://bmjopen.bmj.com/site/about/guidelines.xhtml

prescribed according to participant feedback and task performance as assessed by the treating physiotherapist.

This study will compare acceptability of the HOLOBalance tele-health programme (e.g. compliance, drop-out rate) to an established home exercise programme that is routinely used in community rehabilitation (the OTAGO HEP [23]) and will explore trends for effectiveness across a number of validated outcome measures to explore whether a future trial is warranted, and if so to provide data for a sample size estimate. For this proof of concept study, data will be collected at baseline (week 0) and at completion of the intervention (week 9). The flow of participants through the trial will be recorded in compliance with the CONSORT statement (figure 1), and this protocol has been developed using the SPIRIT reporting guidelines for clinical trials [48].

Participants

One hundred and twenty independently living, community dwelling older adults aged 65-80 who are at risk of falls will be recruited across three sites (London (UK), Freiberg (Germany), Athens (Greece)). Older adults who meet the following inclusion criteria for entry will be eligible to be enrolled.

Inclusion Criteria:

- Independent community-dwelling participants able to walk 500 meters independently or with a stick

- No significant visual impairment

- Able to understand and to consent to the research

- A score of >22 on the MoCA, i.e. adults with no or mild cognitive impairment;

- At risk of falls (i.e. FGA less than 22/30), and / or have significant fear of falling (FESI short form >10) and /or have experienced a fall/s in the last 12 months

- Willing to participate and to comply with the proposed training and testing regime.

- Available space of 1x2 metres at home and sufficient home broadband to allow the system to operate as designed

For peer review only - http://bmjopen.bmj.com/site/about/guidelines.xhtml

Exclusion Criteria:

- Orthostatic hypotension or uncontrolled hypertension

- Have depression i.e. a score of >10 at the Geriatric depression scale

- Have cognitive impairment as indicated by the MoCA score(score <22)

- Other neurological problem (e.g. stroke, Parkinson's disease, peripheral neuropathy)

- Acute musculo-skeletal injury that prevents participation in a structured exercise programme (e.g. lower limb fracture)

- No internet connection at home

- Has participated in a clinical drug trial in the past 6 months

- Currently receiving falls and/or cognitive rehabilitation.

- Has an implanted medical device or cardiac pacemaker

Recruitment

Community dwelling older adults will be recruited via adverts placed in the community, on social media, and by referrals made by recruiting clinics. Potential participants will undergo a telephone screening session with a researcher to determine their eligibility, with eligible participants booked in for baseline testing with the blinded outcome assessor. Participants will provide written informed consent on arrival at their baseline outcome assessment session.

Randomisation

Patients will be randomized using an online platform (www.sealedenvelope.com) into either the intervention or control groups. Participants will be allocated in blocks of 10 with an allocation ratio of 1:1 to control (OTAGO HEP) and intervention (HOLOBalance) groups. Randomisation procedures will be run independently for each site and will be completed by a researcher external to the research study. No out of hours randomisation is required for this study.

Allocation will be concealed in consecutively numbered opaque envelopes which will be drawn up for each block and presented to the treating physiotherapist after the baseline assessment session has been completed. Allocation will not be revealed until after the

For peer review only - http://bmjopen.bmj.com/site/about/guidelines.xhtml

baseline assessment has been completed, and once revealed will be entered onto the enrolment log.

Blinding

This is a single (assessor) blinded proof of concept study. As this study is comparing a technology intervention (HOLOBalance) vs standard care it is not possible to blind the participants or the treating clinicians to the intervention provided. The blinded outcome assessor will collect all of the outcome measures at baseline and follow up with a single assessor being used at each site. Each assessor will be required to attend a training session prior to data collection to ensure consistency across sites. The outcome assessor will be asked to record any incidences of unblinding and detail how this occurred. Due to the nature of this study, we do not foresee circumstances where emergency unblinding of the outcome assessor is required.

Interventions

Participants will be randomised to receive either the active intervention (HOLOBalance) or the control intervention (OTAGO HEP). Participants will be asked to perform their prescribed exercises daily, with each participant performing 40-60 minutes of rehabilitation per day (including rest breaks). Participants will receive home visits from their treating clinician at week 0, 3 and 6 to assess and progress their programme. All participants will have a telephone conversation with their treating clinician every week for the duration of their participation in the rehabilitation programme to discuss their exercise programme.

Active Intervention: The HOLOBalance System

HOLOBalance is an augmented reality balance training programme based upon evidence-based multi-sensory rehabilitation (MSR) protocols [49, 50]. MSR requires individuals to regularly perform exercises which challenge the balance system (e.g. closed eyes, stand on foam), optimise vestibular balance function and have been shown to improve balance control in healthy older adults [49, 50] and people with vestibular balance dysfunction [51-54].

Participants will be required to wear a series of body worn sensors (pressure detecting insoles, accelerometer and a heart rate monitor, Figure 2) when performing the exercises so that 1) the computer software can assess the participants performance and 2) the user can interact with the system. HOLOBalance will provide prescribed exercises and exergames

For peer review only - http://bmjopen.bmj.com/site/about/guidelines.xhtml

presented into the person's home environment by ahead mounted augmented reality display and log the user's interactions with the system (e.g. number of sessions completed). HOLOBalance will display a holographic virtual physiotherapist (Figure 3A) to provide instructions and demonstrate prescribed exercises. The HOLOBalance programme will include exercises to maximise opportunities for balance improvement and include turning the head whilst looking at a target, standing on foam, and walking whilst turning the head (for examples of gameplay please see figure 3B and 3C, and for table of basic exercises please see Table 1). In addition, participants will be given auditory and cognitive exercises in supplement to their balance training programme. For further information on the HOLOBalance intervention, please visit www.holobalance.eu.

Control Intervention: The OTAGO Home Exercise Programme

The OTAGO HEP booklet (available at

https://www.livestronger.org.nz/assets/Uploads/acc1162-otago-exercise-manual.pdf) will be

provided to all individuals in the control arm. Following the physiotherapy home assessment, participants will be provided with a list of exercises from the OTAGO booklet for them to complete every day. Participants will be required to log their exercises in an exercise diary for review by the treating clinician at the next home visit. Exercises will be progressed in line with the recommendations of the OTAGO HEP in combination with the treating clinician's clinical judgement.

Outcome Measures

Primary Outcomes

The primary outcomes will relate to the acceptability, safety and feasibility of providing the HOLOBalance intervention. Acceptability measures include; recruitment rate (percentage of eligible participants enrolled), compliance with interventions (percentage of prescribed sessions completed in HOLOBalance and control groups) and drop-out rates (number of drops outs in HOLOBalance and control groups). Exit interviews will be completed with participants that receive the HOLOBalance intervention to explore their experience of using the system, including perceived benefits, frustrations, and recommendations. Interviews will be recorded, transcribed verbatim and subject to thematic analysis.

For peer review only - http://bmjopen.bmj.com/site/about/guidelines.xhtml

In the HOLOBalance group, adherence will automatically be monitored through the interaction with the system, and calculated as the number of prescribed balance exercises, auditory and cognitive training tasks completed per day, expressed as a percentage. In the control group participants will be required to complete a daily exercise diary to report on their level of activity, whether they had completed their Home Exercise Programme session and whether they had performed their cognitive tasks. All participants will receive a weekly telephone call from the research physiotherapist to discuss any issues, their current progress and to remind them to complete their exercise diaries (if in the control group) which will be collected at their final outcome measure test session. The order of events for participants enrolled in this study is displayed in Table 2.

Safety of the HOLOBalance intervention will be assessed by recording and assessing all adverse / serious adverse events and device effects (and reporting these to appropriate agencies as required). These will be reported by participants to their treating clinician or by self-reporting on the HOLOBalance database. The feasibility of the study will be explored by collecting information on and analysing any deviations from protocol or problems with implementing the study protocol (e.g. logistical problems).

Secondary outcomes

Balance Assessment

The mini BESTest [55] is a 14-item test that assesses dynamic balance components including anticipatory postural adjustments, reactive postural control, sensory orientation and dynamic gait. The data is based on a total score of 28 points. The test takes approximately 10 minutes to complete. This will be collected at baseline (week 0) and follow up (week 9)

The Functional Gait Assessment (FGA) [4] is a 10-item test that assesses performance on complex gait tasks (e.g. walking with head turns or stopping and turning). The test takes approximately 5 minutes to complete. Scores of 21 or less indicate risk for falls [4]. This will be collected at baseline (week 0) and follow up (week 9)

Falls diaries will be used to record whether participants have fallen each day, with falls defined as a person coming to rest inadvertently on the ground or floor or other lower level. Participants will return falls diaries monthly for the duration of the intervention and for 6 months after completion.

Cognitive Assessment

For peer review only - http://bmjopen.bmj.com/site/about/guidelines.xhtml

The validated Montreal Cognitive Assessment (MoCA) [56]: This test includes sections on visuospatial/executive function, naming, attention, language, abstraction, and orientation to time and place. This test will be used as a screening test for cognitive function prior to inclusion in the trial. This will be collected at baseline (week 0) and follow up (week 9).

The validated Cambridge Neuropsychological Test Automated Battery [57]; (CANTAB) cognitive test battery will be used to assess specific cognitive function domains pre-post intervention. The test battery includes: (i) Motor screening task, (ii) Paired Associated Learning, (iii) Spatial Working Memory, (iv) Reaction Time, (v) Rapid Visual Information Processing and (vi) Delayed Matching to Sample. This assessment will take approximately 35 minutes to complete. Processing speed and executive functions from the CANTAB suite (http://www.cambridgecognition.com/academic/cantabsuite/battery) will be compiled to derive an executive function score. This will be collected at baseline (week 0) and follow up (week 9)

Physical Activity and Social Participation Assessment

The Rapid Assessment of Physical Activity [58] (RAPA) is a 9-item, self-administered questionnaire developed to provide an easily administered and interpreted means of assessing levels of physical activity among adults older than 50 years. RAPA evaluates a wide range of physical activity level, from sedentary to vigorous activity, as well as strength and flexibility training and takes 5-minutes to complete. It has adequate convergent validity and good criterion validity. This will be collected at baseline (week 0) and follow up (week8)

The World Health Organization Disability Assessment Schedule 2.0 [59] (WHODAS 2.0) is an assessment which provides a global measure of disability. It covers the following domains of functioning: Cognition – understanding & communicating; Mobility– moving & getting around; Self-care– hygiene, dressing, eating & staying alone; Getting along– interacting with other people; Life activities– domestic responsibilities, leisure, work & school; Participation– joining in community activities. This will be collected at baseline (week 0) and follow up (week 9)

Subjective Questionnaires

The Activities-specific Balance Confidence Scale [60] (ABC) assesses patient's perceived confidence for performing 16-activities of daily living without losing balance. Scores

For peer review only - http://bmjopen.bmj.com/site/about/guidelines.xhtml

≤67/100% indicate increased falls risk. This will be collected at baseline (week 0) and follow up (week 9)

The Falls Efficacy Scale International (FES-I) Short Form is a short, easy to administer tool measuring an individual's level of concern regarding falling during social and physical activities inside and outside the home, whether or not the person actually does the activity. Level of concern is measured on a four-point Likert scale (1=not at all to 4=very). It has excellent internal validity and test-retest reliability. Scores >10 for the short form have been suggested as cut points for indicating high concern about falling [61]. This will be collected at baseline (week 0) and follow up (week 9)

The EQ-5D [62] is a standardized, valid and reliable simple, generic measure of health status for clinical and economic appraisal. The EQ-5D-5L has 5 dimensions (mobility, selfcare, usual activities, pain/discomfort, anxiety/depression) and includes the EQ visual Analogue scale (EQ VAS). The respondent is asked to rate their health status on these five dimensions from 1 to 5 respectively as no problems, slight problems, moderate problems, severe problems, and extreme problems. The EQ VAS records the respondent's self-rated health on a 20 cm vertical, visual analogue scale with endpoints labelled 'the best health you can imagine' and 'the worst health you can imagine'. The respondent is asked to mark an X on the scale to indicate "how your health is TODAY". This will be collected at baseline (week 0) and follow up (week 9)

Environmental Analysis of Mobility [63] (EMS) scale is a self-report scale assessing the effect of the physical environment on community mobility. Twenty-four features of the physical environment are identified. For each feature, an encounter question (How often do you?) is paired with an avoidance question (How often do you avoid?). Subjects report on frequency of encounter and avoidance behaviour using a five-point ordinal scale (never, rarely, sometimes, often, and always). The test-retest reliability of the questionnaire is good. This will be collected at baseline (week 0) and follow up (week 9)

The behavioural regulation in exercise questionnaire (BREQ-3) [64, 65] is a 24 item questionnaire to assess motivation to exercise. Participants rate whether statements apply to themselves (or not) using a 5 point Likert scale ranging from 0 (Not true for me) to 4 (Very true for me). This will be collected at baseline (week 0) and follow up (week 9)

User Experience Evaluation (HOLOBalance Group Only)

The system usability scale (SUS) [66] is a 10 item questionnaire that asks individuals to rate their experience of using the system on a 5 point Likert scale ranging from 1 (strongly

For peer review only - http://bmjopen.bmj.com/site/about/guidelines.xhtml

disagree) to 5 (strongly agree). Example items include ‘I think that I wold like to use this product regularly’. This will be collected by the treating physiotherapist at week 6 only

The user experience questionnaire (UEQ) [67] is a 26 item self-report questionnaire to assess a person’s experience of using a product. Participants rate the product between two contrasting attributes (e.g. attractive vs unattractive) on a 7 point Likert scale. This will be collected by the treating physiotherapist at weeks 1,3 and 6.

The NASA Task Load Index (NASA-TLX)[68] is a widely used, subjective, multidimensional assessment tool that rates perceived workload in order to assess a task, system, or team's effectiveness or other aspects of performance. The NASA TLX assesses work load on five 21 point visual analogue scales ranging from 0 (Very Low) to 21 (Very high) for task demands including mental demand, physical demand and temporal demand. Questions from the NASA TLX will be integrated into the HOLOBalance tasks. These questions will be administered 20 times per participant in total

Data management and statistical analysis

Anonymised data will be entered into a study database. The integrity of this data will be assessed by taking a random sample (10%) from the study database (after the completion of data collection) and comparing with the paper CRF. If there is less than 98% agreement between the paper CRF record and inputted measures, all data will be checked and rectified. The rectified database will be saved under a new filename (e.g. studydatabase_rectified_date) and all changes made to the database will be logged. Essential trial documentation will be kept with the Trial Master File and Investigator Site Files. The sponsor will ensure that study documentation is retained in accordance with their local approvals after the conclusion of the trial.

As this is a proof of concept study, there is no formal plan for statistical analysis due to the small sample size and lack of statistical power. However, for a future trial an analysis plan has been developed which will be run in this study to determine its appropriateness. We will use intention to treat analysis with repeated measures general linear model analysis of variance (ANOVA) for selected outcome measures and other appropriate tests for between-group and within group analysis. We will also include correlations analysis and chi-square assumption testing.

For peer review only - http://bmjopen.bmj.com/site/about/guidelines.xhtml

Sample Size

This proof of concept study will recruit 60 participants per group (i.e. total sample size of 120). This will allow the researchers to gain preliminary data on the primary outcomes of safety, feasibility and acceptability of the HOLOBalance intervention. This will also provide sufficient data to explore trends for effectiveness and to allow for sample size estimates to be drawn up for a future trial.

Study Oversight

Trial steering committees (TSC) and Trial Management Groups (TMG) will be formed to oversee the conduct of the research according to predetermined terms of reference.

Patient Participant Involvement (PPI)

As part of the larger HOLOBalance project, the research consortium has performed extensive PPI with older adults, discussing the study design, exergames and the supporting technologies with approximately 75 older adults in total.

ETHICS AND DISSEMINATION

This study has received institutional ethical approvals in Germany (reference: 265/19) and Greece (reference: 9769/24-6-2019) and from the Health Research Authority in the UK (reference: 19/LO/1908). All amendments to the study protocol which may impact on the integrity of the study, or the data are required to receive approval by the research ethics committee (REC) prior to their implementation.

Findings from this study will be provided as lay reports to participants, disseminated to community groups via community partners and will be submitted for peer reviewed publications. Additionally, electronic data will be anonymised and uploaded to a data repository (Zenodo) that supports restricted access. Electronic data will not be made publicly available and access will to the dataset will only be provided by the data management board of the HOLOBalance research consortium. Use and re-use of the pilot dataset will be subject to the license under which the data objects were deposited.

For peer review only - http://bmjopen.bmj.com/site/about/guidelines.xhtml

TRIAL REGISTRATION

This study has been prospectively registered on www.clinicaltrials.gov (identifier: NCT04053829).

TRIAL STATUS

This study commenced recruitment in April 2020

FUNDING STATEMENT

This project has received funding from the European Union's Horizon 2020 research and innovation programme under grant agreement No 769574. This article reflects only the author's view and the Commission is not responsible for any use that may be made of the information it contains

AUTHOR CONTRIBUTIONS

Matthew B Liston[1], Gregory J Genna[2], Christoph Maurer[3], Dimitris Kikidis[4], Dimitris Gatsios[5,6,] Dimitris Fotiadis, Doris Eva-Bamiou[2,7,8], Marousa Pavlou[1].

MBL, GG, DEB, MP were involved in the conception, design and writing and editing of the study protocol. DG, DF, DK and CM were involved in the conception and editing of the protocol. All authors approved the final protocol.

COMPETING INTERESTS

None

For peer review only - http://bmjopen.bmj.com/site/about/guidelines.xhtml

TABLES

Position	Description	Progressions	Progression Activation
**Seated**	Head turns side to side (yaw rotation of 30 degrees) whilst visually fixating on a static target placed at eye level in front of the participant	Increase speed of movements<br><br>Visual target moves in opposite direction to head movement	Increase speed of head turns if patient reporting no symptoms<br><br>If patient can perform 3 head turns (i.e. left to right) in one second without provoking symptoms, move to next progression
**Seated**	Had turns up and down (pitch rotation of 30 degrees) whilst visually fixating on a static target placed at eye level in front of the participant	Increase speed of movements<br><br>Visual target moves in opposite direction to head movement<br><br>Look up to ceiling and down to floor without fixation	Increase speed of head turns if patient reporting no symptoms<br><br>If patient can perform 3 head turns (i.e. left to right) in one second without provoking symptoms, move to next progression

For peer review only

**Seated**	Bend down to pick object up from the floor in front of you	Close the eyes<br><br>Reach to pick up objects to the side	If patient can complete the full range of movement consistently in 2 seconds (or less) in eyes open condition, and no symptoms are provoked. Progress to eyes closed.<br><br>When able to complete forward bends with eyes open and closed, progress to sideways bend.
**Standing**	Stand with feet hip width apart, looking straight ahead with eyes open	Bring feet closer together<br><br>Close the eyes	If patient can maintain stability in the correct position for one minute with eyes open without prompts, then ask patient to close eyes.<br><br>If patient can maintain stability in the correct position with eyes closed, reduce base of support.

For peer review only - http://bmjopen.bmj.com/site/about/guidelines.xhtml

**Standing**	Stand on foam cushion with feet hip width apart and looking straight ahead with eyes open	Bring feet closer together<br><br>Close the eyes	If patient can maintain stability in the correct position for one minute with eyes open without prompts, then ask patient to close eyes.<br><br>If patient can maintain stability in the correct position with eyes closed, reduce base of support.
**Standing**	Stand with feet hip width apart and bend over to pick up an object from the floor	Bring feet closer together<br><br>Reach up as if to reach into a cupboard	When patient can safely complete full movement in less than 3 seconds, and this produces no symptoms, ask patient to increase speed.<br><br>When patient can safely complete the full movement (without symptoms) in under 2 seconds, move on to the next exercise.

For peer review only - http://bmjopen.bmj.com/site/about/guidelines.xhtml

**Standing**	Step turn through 180° to face the opposite direction	Increase speed of movement	Once patient is able to perform the exercise safely and confidently at their baseline speed, ask them to increase their speed.
**Walking**	Walk forwards looking straight ahead	Increase walking speed	Once patient is able to perform the exercise safely and confidently at their baseline speed with no deviation, ask them to increase their gait speed.
**Walking**	Walk forwards across the room looking between two targets placed at eye level, 1.5 metres apart on the horizontal plane.	Increase walking speed<br><br>Increase head turn speed<br><br>Turn head fully to side as if crossing the street.	Once patient is able to perform the exercise safely and confidently at their baseline speed with no symptoms or deviation, ask them to increase their gait speed.<br><br>Once patient is able to perform the exercise safely and confidently with no symptoms or

For peer review only - http://bmjopen.bmj.com/site/about/guidelines.xhtml

			deviation, ask them to increase their head turn speed<br><br>Once patient is able to perform the exercise safely and confidently with no symptoms or deviation, ask them to increase amplitude of head turns
**Walking**	Walk across the room and nod your head to look up to the ceiling and down to the ground.	Increase walking speed<br><br>Integrate diagonal head movements	Once patient is able to perform the exercise safely and confidently at their baseline speed with no symptoms or deviation, ask them to increase their gait speed.<br><br>Once patient is able to perform the exercise safely and confidently with no symptoms or deviation, ask them to increase their head turn speed<br><br>Once patient is able to perform the exercise safely and confidently with no symptoms or deviation, ask them to integrate diagonal head movements

Table 1: The HOLOBalance intervention basic exercises and their progressions

For peer review only

		Screening Session	Baseline Test Session	Week 0	Daily	Weekly	Monthly	Week 3	Week 6	Follow-Up Test Session	Week 9
Recruitment Procedures	Provide Patient information Sheet	X									
	Provide informed consent		X								
University-based data collection	Demographics		X								
	The MOCA		X							X	
	The RAPA		X							X	
	The WHODAS 2.0		X							X	
	The CANTAB		X							X	
	The ABC Scale		X							X	
	The FES-I		X							X	
	The EQ5D		X							X	
	The EMA		X							X	
	The BREQ -3		X							X	
	Mini-BESTest		X							X	
	The FGA		X							X	
	Exit Interviews (HOLOBalance Group Only)										X
Usability Measures	The SUS								X		
	The UEQ			X				X	X		

For peer review only

**Home-based interactions**	Equipment installation & Removal (HOLOBalance Only)			X						X	
	Home visit from Physiotherapist			X				X	X		
	Telephone conversation with physiotherapist					X					
	Perform Home Exercise Programme				X						
	Submit falls diaries						X				

Table 2: Order of events for all participants enrolled in the HOLOBalance study.

For peer review only

For peer review only - http://bmjopen.bmj.com/site/about/guidelines.xhtml

REFERENCES

1. Scuffham, P., S. Chaplin, and R. Legood, *Incidence and costs of unintentional falls in older people in the United Kingdom.* J Epidemiol Community Health, 2003. **57**(9): p. 740-4.
2. Tinetti, M.E., M. Speechley, and S.F. Ginter, *Risk factors for falls among elderly persons living in the community.* N Engl J Med, 1988. **319**(26): p. 1701-7.
3. Deandrea, S., et al., *Risk factors for falls in community-dwelling older people: a systematic review and meta-analysis.* Epidemiology, 2010. **21**(5): p. 658-68.
4. Wrisley, D.M. and N.A. Kumar, *Functional gait assessment: concurrent, discriminative, and predictive validity in community-dwelling older adults.* Phys Ther, 2010. **90**(5): p. 761-73.
5. Horak, F.B., *Postural orientation and equilibrium: what do we need to know about neural control of balance to prevent falls?* Age Ageing, 2006. **35 Suppl 2**: p. ii7-ii11.
6. Agrawal, Y., et al., *Disorders of balance and vestibular function in US adults: data from the National Health and Nutrition Examination Survey, 2001-2004.* Arch Intern Med, 2009. **169**(10): p. 938-44.
7. Lord, S.R. and R.D. Clark, *Simple physiological and clinical tests for the accurate prediction of falling in older people.* Gerontology, 1996. **42**(4): p. 199-203.
8. Jacobson, G.P., et al., *Significant vestibular system impairment is common in a cohort of elderly patients referred for assessment of falls risk.* J Am Acad Audiol, 2008. **19**(10): p. 799-807.
9. Liston, M.B., et al., *Peripheral vestibular dysfunction is prevalent in older adults experiencing multiple non-syncopal falls versus age-matched non-fallers: a pilot study.* Age Ageing, 2014. **43**(1): p. 38-43.
10. Agrawal, Y., *Editorial: Age-Related Vestibular Loss: Current Understanding and Future Research Directions.* Front Neurol, 2017. **8**: p. 443.
11. Baldursdottir, B., et al., *Sensory impairments and wrist fractures: A case-control study.* J Rehabil Med, 2018. **50**(2): p. 209-215.
12. Kristinsdottir, E.K., G.B. Jarnlo, and M. Magnusson, *Asymmetric vestibular function in the elderly might be a significant contributor to hip fractures.* Scand J Rehabil Med, 2000. **32**(2): p. 56-60.
13. Kristinsdottir, E.K., et al., *Observation of vestibular asymmetry in a majority of patients over 50 years with fall-related wrist fractures.* Acta Otolaryngol, 2001. **121**(4): p. 481-5.
14. Ble, A., et al., *Executive function correlates with walking speed in older persons: the InCHIANTI study.* J Am Geriatr Soc, 2005. **53**(3): p. 410-5.
15. Coppin, A.K., et al., *Association of executive function and performance of dual-task physical tests among older adults: analyses from the InChianti study.* Age Ageing, 2006. **35**(6): p. 619-24.
16. Kearney, F.C., et al., *The relationship between executive function and falls and gait abnormalities in older adults: a systematic review.* Dement Geriatr Cogn Disord, 2013. **36**(1-2): p. 20-35.
17. Liu-Ambrose, T., et al., *Dual-task gait performance among community-dwelling senior women: the role of balance confidence and executive functions.* J Gerontol A Biol Sci Med Sci, 2009. **64**(9): p. 975-82.
18. Rossi, A.F., et al., *The prefrontal cortex and the executive control of attention.* Experimental brain research. Experimentelle Hirnforschung. Experimentation cerebrale, 2009. **192**(3): p. 489-497.
19. Vaughan, L. and K. Giovanello, *Executive function in daily life: Age-related influences of executive processes on instrumental activities of daily living.* Psychol Aging, 2010. **25**(2): p. 343-55.

For peer review only - http://bmjopen.bmj.com/site/about/guidelines.xhtml

20. Yogev-Seligmann, G., J.M. Hausdorff, and N. Giladi, *The role of executive function and attention in gait.* Mov Disord, 2008. **23**(3): p. 329-42; quiz 472.
21. Boyle, P.A., et al., *Executive dysfunction and apathy predict functional impairment in Alzheimer disease.* Am J Geriatr Psychiatry, 2003. **11**(2): p. 214-21.
22. Muir, S.W., K. Gopaul, and M.M. Montero Odasso, *The role of cognitive impairment in fall risk among older adults: a systematic review and meta-analysis.* 2012. **41**(3): p. 299-308.
23. Delbaere, K., et al., *Mild cognitive impairment as a predictor of falls in community-dwelling older people.* Am J Geriatr Psychiatry, 2012. **20**(10): p. 845-53.
24. Bridenbaugh, S.A. and R.W. Kressig, *Motor cognitive dual tasking.* 2015. **48**(1): p. 15-21.
25. Frewen, J., et al., *Recent syncope and unexplained falls are associated with poor cognitive performance.* Age Ageing, 2015. **44**(2): p. 282-286.
26. Dommes, A. and V. Cavallo, *The role of perceptual, cognitive, and motor abilities in street-crossing decisions of young and older pedestrians.* Ophthalmic Physiol Opt, 2011. **31**(3): p. 292-301.
27. World Health Organization. *Physical Activity*. 2018; Available from: https://www.who.int/news-room/fact-sheets/detail/physical-activity.
28. Public Health England, *Falls and Fracture consensus statement*. 2017, Public Health England.
29. Public Health England. *Physical Activity: applying All Our Health*. 2019 20-12-2019]; Available from: https://www.gov.uk/government/publications/physical-activity-applying-all-our-health/physical-activity-applying-all-our-health.
30. National Institute for Heath and Care Excellence, *Falls in Older People: Assessing Risk and Prevention (CG161)*. 2013, NICE: UK.
31. Physicians, R.C.o., *Falling standards broken promises: Report of the national audit of falls and bone health in older people 2010*. 2011, Royal College of Physicians.
32. Sherrington, C., et al., *Exercise to prevent falls in older adults: an updated systematic review and meta-analysis.* Br J Sports Med, 2017. **51**(24): p. 1750-1758.
33. Hall, C.D., et al., *Vestibular Rehabilitation for Peripheral Vestibular Hypofunction: An Evidence-Based Clinical Practice Guideline: FROM THE AMERICAN PHYSICAL THERAPY ASSOCIATION NEUROLOGY SECTION.* J Neurol Phys Ther, 2016. **40**(2): p. 124-55.
34. Sherrington, C., et al., *Exercise for preventing falls in older people living in the community: an abridged Cochrane systematic Review.* British Journal of Sports Medicine, 2019: p. bjsports-2019-1.
35. Pavlou, M., A.M. Bronstein, and R.A. Davies, *Randomized trial of supervised versus unsupervised optokinetic exercise in persons with peripheral vestibular disorders.* Neurorehabil Neural Repair, 2013. **27**(3): p. 208-18.
36. Campbell, A.J., et al., *Falls prevention over 2 years: a randomized controlled trial in women 80 years and older.* Age Ageing, 1999. **28**(6): p. 513-8.
37. Robertson, M.C., et al., *Preventing injuries in older people by preventing falls: a meta-analysis of individual-level data.* J Am Geriatr Soc, 2002. **50**(5): p. 905-11.
38. Robertson, M.C., et al., *Effectiveness and economic evaluation of a nurse delivered home exercise programme to prevent falls. 1: Randomised controlled trial.* BMJ, 2001. **322**(7288): p. 697-701.
39. Robertson, M.C., et al., *Effectiveness and economic evaluation of a nurse delivered home exercise programme to prevent falls. 2: Controlled trial in multiple centres.* BMJ, 2001. **322**(7288): p. 701-4.
40. Skelton, D. and S. Dinan, *Exercise for falls management: Rationale for an exercise programme aimed at reducing postural instability.* Physiotherapy Theory and Practise, 1999. **15**: p. 105-20.
41. Skelton, D., et al., *Tailored group exercise (Falls Management Exercise -- FaME) reduces falls in community-dwelling older frequent fallers (an RCT).* Age Ageing, 2005. **34**(6): p. 636-9.

For peer review only - http://bmjopen.bmj.com/site/about/guidelines.xhtml

42. Falbo, S., et al., *Effects of Physical-Cognitive Dual Task Training on Executive Function and Gait Performance in Older Adults: A Randomized Controlled Trial.* BioMed Research International, 2016. **2016**: p. 1-12.
43. Silsupadol, P., et al., *Training-related changes in dual-task walking performance of elderly persons with balance impairment: a double-blind, randomized controlled trial.* Gait Posture, 2009. **29**(4): p. 634-9.
44. Wollesen, B., et al., *Effects of Dual-Task Management and Resistance Training on Gait Performance in Older Individuals: A Randomized Controlled Trial.* Frontiers in Aging Neuroscience, 2017. **9**.
45. Liston, M.B., et al., *Feasibility and effect of supplementing a modified OTAGO intervention with multisensory balance exercises in older people who fall: a pilot randomized controlled trial.* Clin Rehabil, 2014. **28**(8): p. 784-793.
46. Baldursdottir, B., et al., *Multi-sensory training and wrist fractures: a randomized, controlled trial.* Aging Clinical and Experimental Research, 2019.
47. Kristinsdottir, E.K. and B. Baldursdottir, *Effect of multi-sensory balance training for unsteady elderly people: pilot study of the "Reykjavik model".* Disability and rehabilitation, 2014. **36**(14): p. 1211-1218.
48. Chan, A.-W., et al., *SPIRIT 2013 Statement: Defining Standard Protocol Items for Clinical Trials.* Annals of Internal Medicine, 2013. **158**(3): p. 200.
49. Hu, M.H. and M.H. Woollacott, *Multisensory training of standing balance in older adults: II. Kinematic and electromyographic postural responses.* J Gerontol, 1994. **49**(2): p. M62-71.
50. Hu, M.H. and M.H. Woollacott, *Multisensory training of standing balance in older adults: I. Postural stability and one-leg stance balance.* J Gerontol, 1994. **49**(2): p. M52-61.
51. Shumway-Cook, A. and F.B. Horak, *Rehabilitation strategies for patients with vestibular deficits.* Neurol Clin, 1990. **8**(2): p. 441-57.
52. Whitney, S.L., A.A. Alghwiri, and A. Alghadir, *An overview of vestibular rehabilitation.* Handb Clin Neurol, 2016. **137**: p. 187-205.
53. Whitney, S.L. and P.J. Sparto, *Principles of vestibular physical therapy rehabilitation.* NeuroRehabilitation, 2011. **29**(2): p. 157-66.
54. Hall, C.D., et al., *Vestibular Rehabilitation for Peripheral Vestibular Hypofunction.* Journal of Neurologic Physical Therapy, 2016. **40**(2): p. 124-155.
55. Franchignoni, F., et al., *Using psychometric techniques to improve the Balance Evaluation Systems Test: the mini-BESTest.* J Rehabil Med, 2010. **42**(4): p. 323-31.
56. Nasreddine, Z.S., et al., *The Montreal Cognitive Assessment, MoCA: a brief screening tool for mild cognitive impairment.* J Am Geriatr Soc, 2005. **53**(4): p. 695-9.
57. Smith, P.J., et al., *A comparison of the Cambridge Automated Neuropsychological Test Battery (CANTAB) with "traditional" neuropsychological testing instruments.* Journal of Clinical and Experimental Neuropsychology, 2013. **35**(3): p. 319-328.
58. Topolski, T.D., et al., *The Rapid Assessment of Physical Activity (RAPA) among older adults.* Preventing chronic disease, 2006. **3**(4): p. A118-A118.
59. Silveira, C., et al., *Validation of the WHO Disability Assessment Schedule (WHODAS 2.0) 12-item tool against the 36-item version for measuring functioning and disability associated with pregnancy and history of severe maternal morbidity.* International Journal of Gynecology & Obstetrics, 2018. **141**: p. 39-47.
60. Powell, L.E. and A.M. Myers, *The Activities-specific Balance Confidence (ABC) Scale.* J Gerontol A Biol Sci Med Sci, 1995. **50A**(1): p. M28-34.
61. Delbaere, K., et al., *The Falls Efficacy Scale International (FES-I). A comprehensive longitudinal validation study.* Age and Ageing, 2010. **39**(2): p. 210-216.
62. *EuroQol - a new facility for the measurement of health-related quality of life.* Health Policy, 1990. **16**(3): p. 199-208.

For peer review only - http://bmjopen.bmj.com/site/about/guidelines.xhtml

63. Shumway-Cook, A., et al., *Assessing Environmentally Determined Mobility Disability: Self-Report Versus Observed Community Mobility.* Journal of the American Geriatrics Society, 2005. **53**(4): p. 700-704.
64. Wilson, P.M., et al., *"It's Who I Am … Really!' The Importance of Integrated Regulation in Exercise Contexts1.* 2007. **11**(2): p. 79-104.
65. Markland, D. and V. Tobin, *A modification to the Behavioural Regulation in Exercise Questionnaire to include an assessment of amotivation.* Journal of Sport & Exercise Psychology, 2004. **26**(2): p. 191-196.
66. Lewis, J.R., *The System Usability Scale: Past, Present, and Future.* International Journal of Human–Computer Interaction, 2018. **34**(7): p. 577-590.
67. Laugwitz, B., T. Held, and M. Schrepp. *Construction and Evaluation of a User Experience Questionnaire*. in *HCI and Usability for Education and Work*. 2008. Berlin, Heidelberg: Springer Berlin Heidelberg.
68. Hart, S.G. and L.E. Staveland, *Development of NASA-TLX (Task Load Index): Results of Empirical and Theoretical Research*, in *Advances in Psychology*, P.A. Hancock and N. Meshkati, Editors. 1988, North-Holland. p. 139-183.

Figure 1: CONSORT diagram to demonstrate participant flow through the HOLOBalance study

Figure 2: Diagram of the HOLOBalance system hardware

Figure 3: Screenshots of (A) physiotherapist avatar, (B and C) representative gameplay from the HOLOBalance system

For peer review only

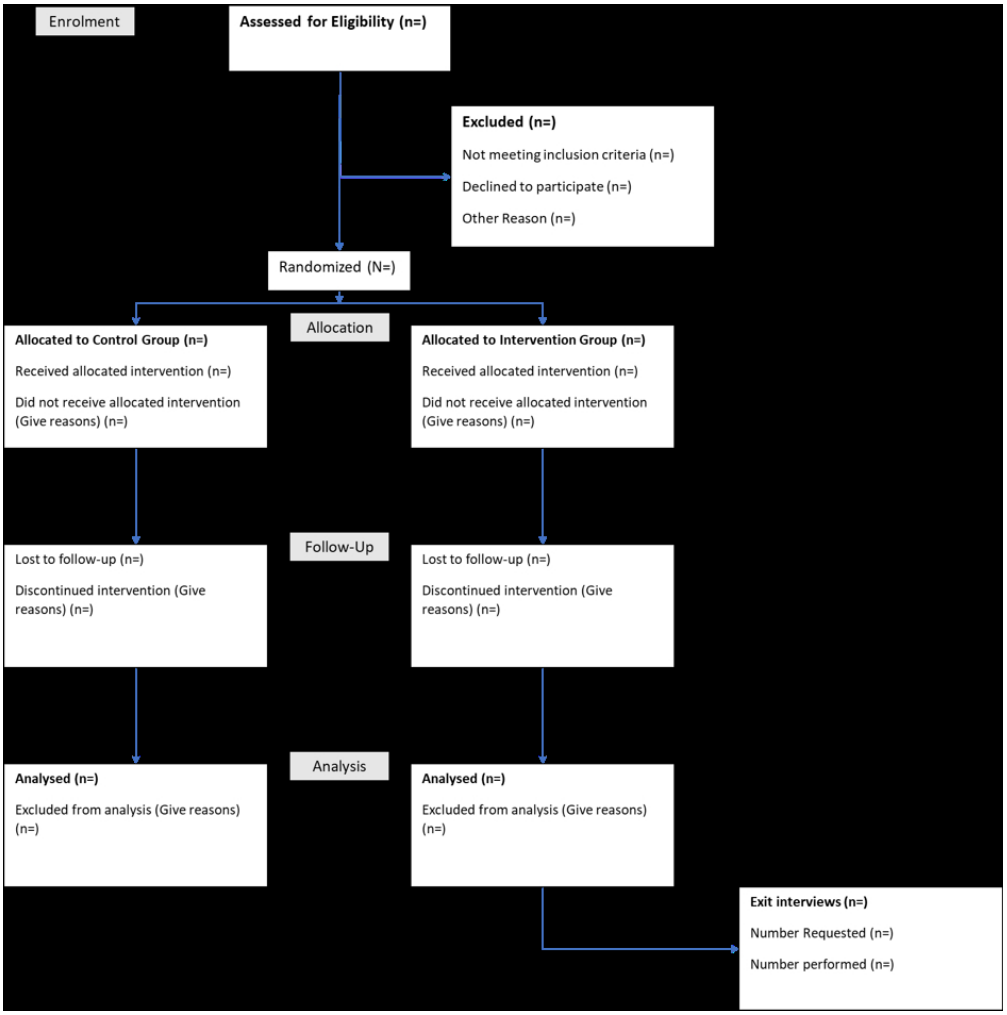

CONSORT diagram to demonstrate participant flow through the HOLOBalance study

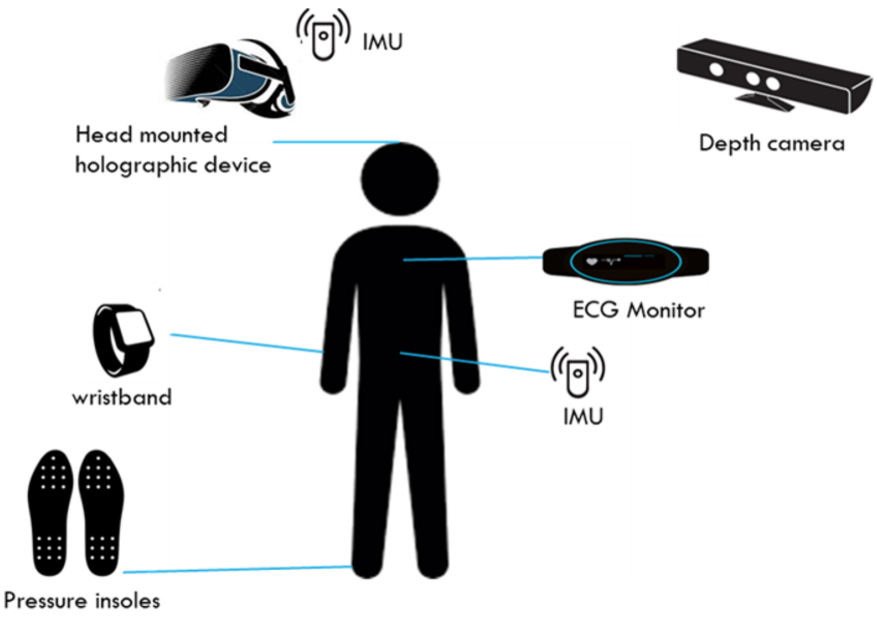

Diagram of the HOLOBalance system hardware

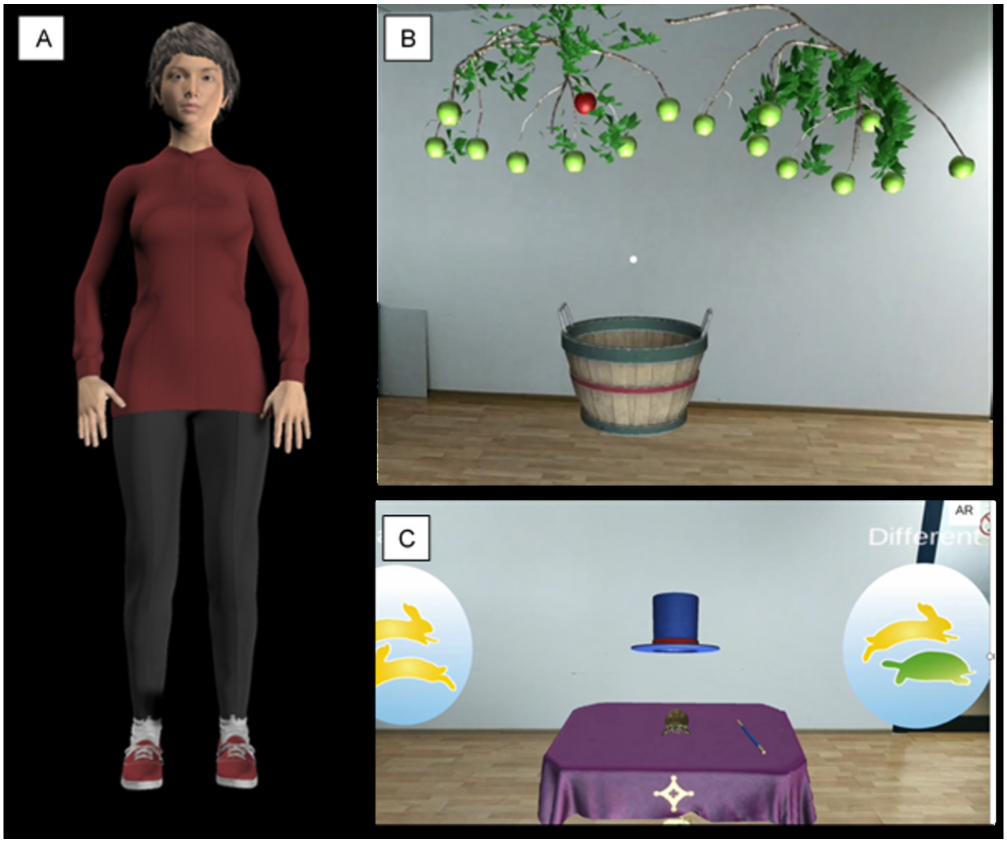

Screenshots of (A) physiotherapist avatar, (B and C) representative gameplay from the HOLOBalance system

Reporting checklist for protocol of a clinical trial.

Based on the SPIRIT guidelines.

Instructions to authors

Complete this checklist by entering the page numbers from your manuscript where readers will find each of the items listed below.

Your article may not currently address all the items on the checklist. Please modify your text to include the missing information. If you are certain that an item does not apply, please write "n/a" and provide a short explanation.

Upload your completed checklist as an extra file when you submit to a journal.

In your methods section, say that you used the SPIRITreporting guidelines, and cite them as:

Chan A-W, Tetzlaff JM, Altman DG, Laupacis A, Gøtzsche PC, Krleža-Jerić K, Hróbjartsson A, Mann H, Dickersin K, Berlin J, Doré C, Parulekar W, Summerskill W, Groves T, Schulz K, Sox H, Rockhold FW, Rennie D, Moher D. SPIRIT 2013 Statement: Defining standard protocol items for clinical trials. Ann Intern Med. 2013;158(3):200-207

		Reporting Item	Page Number
**Administrative information**			
Title	#1	Descriptive title identifying the study design, population, interventions, and, if applicable, trial acronym	1

For peer review only

Trial registration	#2a	Trial identifier and registry name. If not yet registered, name of intended registry	2 and 14
Trial registration: data set	#2b	All items from the World Health Organization Trial Registration Data Set	NA, This information is included in Clinical Trial Registration
Protocol version	#3	Date and version identifier	1
Funding	#4	Sources and types of financial, material, and other support	15
Roles and responsibilities: contributorship	#5a	Names, affiliations, and roles of protocol contributors	1
Roles and responsibilities: sponsor contact information	#5b	Name and contact information for the trial sponsor	15
Roles and responsibilities: sponsor and funder	#5c	Role of study sponsor and funders, if any, in study design; collection, management, analysis, and interpretation of data; writing of the report; and the decision to submit the report for publication, including whether they will have ultimate authority over any of these activities	15

For peer review only

Roles and responsibilities: committees	#5d	Composition, roles, and responsibilities of the coordinating centre, steering committee, endpoint adjudication committee, data management team, and other individuals or groups overseeing the trial, if applicable (see Item 21a for data monitoring committee)	14
**Introduction**			
Background and rationale	#6a	Description of research question and justification for undertaking the trial, including summary of relevant studies (published and unpublished) examining benefits and harms for each intervention	4
Background and rationale: choice of comparators	#6b	Explanation for choice of comparators	6
Objectives	#7	Specific objectives or hypotheses	9
Trial design	#8	Description of trial design including type of trial (eg, parallel group, crossover, factorial, single group), allocation ratio, and framework (eg, superiority, equivalence, non-inferiority, exploratory)	5
**Methods: Participants,**			

For peer review only

**interventions, and outcomes**			
Study setting	#9	Description of study settings (eg, community clinic, academic hospital) and list of countries where data will be collected. Reference to where list of study sites can be obtained	6
Eligibility criteria	#10	Inclusion and exclusion criteria for participants. If applicable, eligibility criteria for study centres and individuals who will perform the interventions (eg, surgeons, psychotherapists)	6,7
Interventions: description	#11a	Interventions for each group with sufficient detail to allow replication, including how and when they will be administered	8,9. Table 1.
Interventions: modifications	#11b	Criteria for discontinuing or modifying allocated interventions for a given trial participant (eg, drug dose change in response to harms, participant request, or improving / worsening disease)	9, Table 1
Interventions: adherance	#11c	Strategies to improve adherence to intervention protocols, and any procedures for monitoring adherence (eg, drug tablet return; laboratory tests)	8,9

For peer review only

Interventions: concomitant care	#11d	Relevant concomitant care and interventions that are permitted or prohibited during the trial	7
Outcomes	#12	Primary, secondary, and other outcomes, including the specific measurement variable (eg, systolic blood pressure), analysis metric (eg, change from baseline, final value, time to event), method of aggregation (eg, median, proportion), and time point for each outcome. Explanation of the clinical relevance of chosen efficacy and harm outcomes is strongly recommended	9-13
Participant timeline	#13	Time schedule of enrolment, interventions (including any run-ins and washouts), assessments, and visits for participants. A schematic diagram is highly recommended (see Figure)	Fig 1, Table 2
Sample size	#14	Estimated number of participants needed to achieve study objectives and how it was determined, including clinical and statistical assumptions supporting any sample size calculations	14
Recruitment	#15	Strategies for achieving adequate participant enrolment to reach target sample size	7

For peer review only

Methods: Assignment of interventions (for controlled trials)

Allocation: sequence generation	#16a	Method of generating the allocation sequence (eg, computer-generated random numbers), and list of any factors for stratification. To reduce predictability of a random sequence, details of any planned restriction (eg, blocking) should be provided in a separate document that is unavailable to those who enrol participants or assign interventions	7
Allocation concealment mechanism	#16b	Mechanism of implementing the allocation sequence (eg, central telephone; sequentially numbered, opaque, sealed envelopes), describing any steps to conceal the sequence until interventions are assigned	7
Allocation: implementation	#16c	Who will generate the allocation sequence, who will enrol participants, and who will assign participants to interventions	8
Blinding (masking)	#17a	Who will be blinded after assignment to interventions (eg, trial participants, care	8

For peer review only

		providers, outcome assessors, data analysts), and how	
Blinding (masking): emergency unblinding	#17b	If blinded, circumstances under which unblinding is permissible, and procedure for revealing a participant's allocated intervention during the trial	8
**Methods: Data collection, management, and analysis**			
Data collection plan	#18a	Plans for assessment and collection of outcome, baseline, and other trial data, including any related processes to promote data quality (eg, duplicate measurements, training of assessors) and a description of study instruments (eg, questionnaires, laboratory tests) along with their reliability and validity, if known. Reference to where data collection forms can be found, if not in the protocol	9-13
Data collection plan: retention	#18b	Plans to promote participant retention and complete follow-up, including list of any outcome data to be collected for participants who discontinue or deviate from intervention protocols	8-9

For peer review only

Data management	#19	Plans for data entry, coding, security, and storage, including any related processes to promote data quality (eg, double data entry; range checks for data values). Reference to where details of data management procedures can be found, if not in the protocol	13
Statistics: outcomes	#20a	Statistical methods for analysing primary and secondary outcomes. Reference to where other details of the statistical analysis plan can be found, if not in the protocol	13
Statistics: additional analyses	#20b	Methods for any additional analyses (eg, subgroup and adjusted analyses)	13
Statistics: analysis population and missing data	#20c	Definition of analysis population relating to protocol non-adherence (eg, as randomised analysis), and any statistical methods to handle missing data (eg, multiple imputation)	13
**Methods: Monitoring**			
Data monitoring: formal committee	#21a	Composition of data monitoring committee (DMC); summary of its role and reporting structure; statement of whether it is independent from the sponsor and competing interests; and reference to where further	NA. This was not deemed as necessary as this is a low risk study with short duration (data

For peer review only

		details about its charter can be found, if not in the protocol. Alternatively, an explanation of why a DMC is not needed	collection less than 12 months)
Data monitoring: interim analysis	#21b	Description of any interim analyses and stopping guidelines, including who will have access to these interim results and make the final decision to terminate the trial	9-10
Harms	#22	Plans for collecting, assessing, reporting, and managing solicited and spontaneously reported adverse events and other unintended effects of trial interventions or trial conduct	9-10
Auditing	#23	Frequency and procedures for auditing trial conduct, if any, and whether the process will be independent from investigators and the sponsor	9
**Ethics and dissemination**			
Research ethics approval	#24	Plans for seeking research ethics committee / institutional review board (REC / IRB) approval	14
Protocol amendments	#25	Plans for communicating important protocol modifications (eg, changes to eligibility criteria, outcomes, analyses) to relevant	14

For peer review only

		parties (eg, investigators, REC / IRBs, trial participants, trial registries, journals, regulators)	
Consent or assent	#26a	Who will obtain informed consent or assent from potential trial participants or authorised surrogates, and how (see Item 32)	7
Consent or assent: ancillary studies	#26b	Additional consent provisions for collection and use of participant data and biological specimens in ancillary studies, if applicable	Appendix 1 (CF)
Confidentiality	#27	How personal information about potential and enrolled participants will be collected, shared, and maintained in order to protect confidentiality before, during, and after the trial	13,14
Declaration of interests	#28	Financial and other competing interests for principal investigators for the overall trial and each study site	15
Data access	#29	Statement of who will have access to the final trial dataset, and disclosure of contractual agreements that limit such access for investigators	14
Ancillary and post trial care	#30	Provisions, if any, for ancillary and post-trial care, and for compensation to those who suffer harm from trial participation	Appendix 2 (PIS)

For peer review only

Dissemination policy: trial results	#31a	Plans for investigators and sponsor to communicate trial results to participants, healthcare professionals, the public, and other relevant groups (eg, via publication, reporting in results databases, or other data sharing arrangements), including any publication restrictions	13
Dissemination policy: authorship	#31b	Authorship eligibility guidelines and any intended use of professional writers	14
Dissemination policy: reproducible research	#31c	Plans, if any, for granting public access to the full protocol, participant-level dataset, and statistical code	14
**Appendices**			
Informed consent materials	#32	Model consent form and other related documentation given to participants and authorised surrogates	Appendix 1 and 2
Biological specimens	#33	Plans for collection, laboratory evaluation, and storage of biological specimens for genetic or molecular analysis in the current trial and for future use in ancillary studies, if applicable	Appendix 1 and 2

None The SPIRIT checklist is distributed under the terms of the Creative Commons Attribution License CC-BY-ND 3.0. This checklist can be completed online using https://www.goodreports.org/, a tool made by the EQUATOR Network in collaboration with Penelope.ai

For peer review only - http://bmjopen.bmj.com/site/about/guidelines.xhtml
